# Genetic identification of three CITES-listed sharks using a paper-based Lab-on-a-Chip (LOC)

**DOI:** 10.1371/journal.pone.0300383

**Published:** 2024-04-04

**Authors:** Guuske P. Tiktak, Alexandria Gabb, Margarita Brandt, Fernando R. Diz, Karla Bravo-Vásquez, César Peñaherrera-Palma, Jonathan Valdiviezo-Rivera, Aaron Carlisle, Louise M. Melling, Bradley Cain, David Megson, Richard Preziosi, Kirsty J. Shaw

**Affiliations:** 1 Ecology & Environment Research Centre, Manchester Metropolitan University, Manchester, United Kingdom; 2 Colegio de Ciencias Biológicas y Ambientales, Universidad San Francisco de Quito (USFQ), Quito, Ecuador; 3 Instituto Biósfera, Universidad San Francisco de Quito (USFQ), Quito, Ecuador; 4 Marine Conservation Program, WWF Fisheries Ecuador, Guayaquil, Ecuador; 5 Plan de Acción Nacional para la Conservación y el Manejo de Tiburones de Ecuador, Viceministerio de Acuacultura y Pesca, Ministerio de Producción, Comercio Exterior, Inversiones y Pesca, Puerto Pesquero Artesanal de San Mateo, Manta, Manabí, Ecuador; 6 MigraMar, Bodega Bay, CA, United States of America; 7 Instituto Nacional de Biodiversidad, Quito, Pichincha, Ecuador; 8 School of Marine Science and Policy, University of Delaware, Lewes, Delaware, United States of America; 9 School of Biological and Marine Sciences, University of Plymouth, Plymouth, United Kingdom; University of Vienna: Universitat Wien, AUSTRIA

## Abstract

Threatened shark species are caught in large numbers by artisanal and commercial fisheries and traded globally. Monitoring both which shark species are caught and sold in fisheries, and the export of CITES-restricted products, are essential in reducing illegal fishing. Current methods for species identification rely on visual examination by experts or DNA barcoding techniques requiring specialist laboratory facilities and trained personnel. The need for specialist equipment and/or input from experts means many markets are currently not monitored. We have developed a paper-based Lab-on-a-Chip (LOC) to facilitate identification of three threatened and CITES-listed sharks, bigeye thresher (*Alopias superciliosus*), pelagic thresher (*A*. *pelagicus*) and shortfin mako shark (*Isurus oxyrinchus*) at market source. DNA was successfully extracted from shark meat and fin samples and combined with DNA amplification and visualisation using Loop Mediated Isothermal Amplification (LAMP) on the LOC. This resulted in the successful identification of the target species of sharks in under an hour, with a working positive and negative control. The LOC provided a simple “yes” or “no” result via a colour change from pink to yellow when one of the target species was present. The LOC serves as proof-of-concept (PoC) for field-based species identification as it does not require specialist facilities. It can be used by non-scientifically trained personnel, especially in areas where there are suspected high frequencies of mislabelling or for the identification of dried shark fins in seizures.

## 1. Introduction

Elasmobranchs (sharks, rays and skates) represent one of the most vulnerable taxa on the planet, where over one third of all elasmobranchs are threatened with extinction [1,2]. One of the primary drivers of decline across the group is overfishing [1,3]. Some species have experienced population declines of over 90%, and without effective conservation strategies many species may go extinct altogether [1,3]. Elasmobranchs often occupy tertiary positions in food chains as meso- and apex predators, playing a crucial role in ecosystem functions [4–6]. Many elasmobranch species exhibit similar life-history traits to that of large mammals with long gestation periods, slow maturity, and low fecundity, which makes them especially vulnerable to overexploitation [7,8].

Shark meat is traded and consumed globally, and there is growing concern for the widespread practice of species mislabelling and substitution, even in non-coastal regions where sharks may not be typically considered a primary food source [9]. Mislabelling and species substitution occurs when sharks are sold as other elasmobranch species or teleost fish, and consumers may thus be unaware that they are consuming shark products [10,11]. Mislabelling can pose a threat to the safety of consumers as they may be exposed to allergens, zoonotic diseases, and high concentrations of pollutants, without their knowledge [12,13]. Countries where mislabelling and species substitution has occurred include the UK, Brazil, Greece, Indonesia, Singapore, Taiwan, Peru, Italy, Spain, and the USA, amongst others [9–11,14–16].

Ecuador is home to a diverse array of shark species, but populations have experienced declines driven by the demand for shark fins and meat in international markets. They have also been traditionally sold and consumed in fish markets along the coast of Ecuador [17]. In 2007, the Ecuadorian government prohibited shark finning and directed shark fisheries in the country to reduce the illegal trade but allowed for the sale of incidentally whole caught sharks (fins and body), except in the Galápagos Marine Reserve where sharks are fully protected (Executive Decree 486 of 2007, cited in [18]). Additional efforts to protect sharks included prohibiting the fishing of hammerhead (Sphyrnidae) and oceanic whitetip sharks (*Carcharhinus longimanus*) (Ministerial Agreement MCEIP-SRP-2020-0084-A, cited in [18]). Despite these efforts, over one million shark landings were reported in Ecuador between 2008 and 2012 with the Convention on International Trade in Endangered Species of Wild Fauna and Flora (CITES)-listed shark species: bigeye thresher (*Alopias superciliosus*), pelagic thresher (*A*. *pelagicus*) and shortfin mako (*Isurus oxyrinchus*) sharks accounting for 61% of the total landings during that period [19]. Two of which are listed as Endangered (pelagic thresher and shortfin mako shark), and one as Vulnerable (bigeye thresher shark) in the IUCN Red List [2].

In addition to monitoring trade in shark products, many studies investigating the ecology, biology and life-history of under-studied elasmobranch species rely directly on artisanal and commercial fisheries in remote areas [20–23], where taxonomic specialists may not be present. This can result in morphologically similar species being misidentified which presents a serious threat to our understanding of these species, which is vital to inform conservation and management strategies [24].

Current monitoring is primarily undertaken using DNA barcoding as a technique to identify sharks when they cannot be identified based on their morphological features. It may also be used to confirm visual identification, for example when shark fins have been processed and dried [25–27]. Many studies use species-specific primers to perform polymerase chain reaction (PCR)-based amplification of DNA from mitochondrial and nuclear genes such as cytochrome b, cytochrome c oxidase subunit I (COI), NADH2 and ITS2, and then visualised through gel electrophoresis and/or DNA sequencing [28–30]. More recently, Loop Mediated Isothermal Amplification (LAMP) has been demonstrated to amplify short DNA fragments from twelve CITES-listed shark species [31,32], not including shortfin makos shark. Although these genetic techniques have become increasingly popular over the past few decades, they remain time-consuming and expensive, especially as they rely heavily on access to costly laboratory facilities, trained personnel, specialised field equipment, and even international export if they are sent to labs in other countries [33,34]. One example of shark identification in the field involves the use of a multiplex PCR mini-barcode assay that can rapidly identify processed shark products at a low cost of $1 [27]. Nevertheless, this method still requires the use of trained personnel and specialistic equipment such as thermocyclers and sequencers. Developments in genetic techniques have allowed scientists to sequence DNA in the field, i.e., using a hand-held sequencing device such as the minION (Nanopore, UK) [35,36], nevertheless it costs approximately $60-$80 per sample to run and requires considerable expertise [37]. Thus, these techniques may not be suitable for implementing in countries where control authorities have limited or no access to these technologies or technical expertise.

Miniaturised laboratory techniques are becoming increasing popular as they are often rapid, cost-effective, and portable (e.g., can be carried out in the field). One example of these techniques is Lab-on-a-Chip (LOC) technology. The LOC incorporates several laboratory processes on a small device that is usually only a few square centimetres in size [38]. LOCs are typically made of glass or polydimethylsiloxane (PDMS), though recent development in the field has incorporated the use of paper-based microfluidic chips, which further decreases the cost of application and can be as low as $0.01 per sample [39,40]. LOCs have primarily been used for clinical diagnostics and biomedical research, for example glucose monitoring for diabetes, COVID-19 detection and HIV [38]. Significant development in this field over the past 15 years has allowed for point-of-care (PoC) diagnostics, though there has been limited use of LOC technology in conservation [41].

We have developed a simple, on-site identification tool in the form of a LOC, which can be easily deployed to monitor the trade of three threatened and CITES-listed sharks: bigeye thresher, pelagic thresher and shortfin mako belonging to the order Lamniformes. This study specifically aimed to 1) develop a field-based cell lysis and DNA extraction method that would be suitable for shark muscle and wet fin tissue samples; 2) design species-specific LAMP primers for the three CITES-listed sharks for visual identification; 3) combine the two previous steps, along with positive and negative controls, into an integrated paper-based LOC device; and 4) evaluate the applicability of the LOC device through proof-of-concept field testing and end-user workshops.

## 2. Materials and methods

### 2.1. Sample collection

For the initial development of the LOC, a total of 31 tissue samples were collected from 26 different species of sharks, rays and fish, and confirmed by Sanger sequencing of the COI gene (see section 2.2. for further details). Eleven were collected from fishing ports and markets across three regions in Ecuador between June and July 2018 (Table 1). The sampling sites included Mercado de Mariscos Santa Rosa of Salinas in the province of Santa Elena (coordinates: 2° 13’ 0" South, 80° 58’ 0" West), Playita Mía in Manta, province of Manabí (coordinates: 0° 57’ 10" South, 80° 48’ 45" West) and Puerto Pesquero Artesanal de Esmeraldas situated in the province of Esmeraldas (coordinates: 0°57’33.12"North, 79°39’14.29" West) [CITES Permit: No. 18EC000025/VS; Contrato Marco: MAE-DNB-CM-2016-0045]. Permits in Ecuador were granted by the Ministerio del Ambiente and were obtained with the Instituto Nacional de Biodiversidad. One fin clip of whale shark (*Rhincodon typus*) was collected from the Galapágos Islands, Ecuador [CITES Permit: No. 18EC000019/VS and 18EC000020/VS; Contrato Marco: MAE-DNB-CM-2016-0041], 17 tissue samples were also received from the USA [UK CITES No. GB040, and U.S.A. CITES No. US044], and opportunistic fin clips were taken from two species of shark from the Sea Life Paris Aquarium. For US samples, collection was undertaken with permits from the California Department of Fish and Game, Monterey Bay National Marine Sanctuary, Gulf of the Farallones National Marine Sanctuary, U. S. National Park Service, Naval Postgraduate School, and under Stanford University animal care protocol 10765. Specific permit numbers: Delaware Division of Fish and Wildlife 2021-FSC-021, California Department of Fish and Wildlife SC-8372, National Oceanic and Atmospheric Administration (NOAA) and Office of National Marine Sanctuaries (ONMS) permit MULTI-2014-013-A3. An additional 12 samples were collected from Ecuador between June and July 2018 (bigeye thresher shark, pelagic thresher shark, shortfin mako shark, and blue shark; *n* = 3 per species) for evaluation of the prototype LOC. These sharks were identified visually and were confirmed by LAMP. No live specimens were involved in the sample collection. All samples were stored in nucleic acid preservation (NAP) buffer, except for aquarium tissue samples, which were stored in 95% ethanol. All samples were kept at room temperature for short-term storage (2 months) and then at -20°C for long-term storage.

**Table 1:** Elasmobranch and teleost fish species collected in Ecuador, USA and in captivity (Aquarium France as well as the percentage (%) match from Sanger Sequencing for both Forward (F) and Reverse (R) sequences for each species.

Common name	Species	Location	Tissue type	F	R
Clearnose skate	*Raja eglanteria*	USA	Muscle	90.4%	89.7%
Sandbar shark	*Carcharhinus plumbeus*	USA	Muscle	99.1%	98.5%
Salmon shark	*Lamna ditropis*	USA	Muscle	NA	97.9%
Shortfin mako (1)	*Isurus oxyrinchus*	USA	Muscle	99.4%	99.3%
Blue shark	*Prionace glauca*	USA	Muscle	91.3%	83.6%
Pelagic stingray	*Pteroplatyrygon violacea*	USA	Muscle	96.7%	97.1%
Common thresher	*Alopias vulpinus*	USA	Muscle	99.7%	98.6%
Great white shark	*Carcharodon carcharias*	USA	Muscle	98.9%	NA
Leopard shark	*Triakis semifasciata*	USA	Muscle	98.9%	99.5%
Bigeye thresher (1)	*Alopias superciliosus*	USA	Muscle	99.7%	88.1%
Kitefin shark	*Dalatis licha*	USA	Muscle	89.9%	89.4%
Black tip shark	*Carcharhinus limbatus*	USA	Muscle	90.4%	NA
Great hammerhead shark	*Sphyrna mokarran*	USA	Muscle	99.0%	99.4%
Scalloped hammerhead (1)	*Sphyrna lewini*	USA	Muscle	98.5%	99.1%
Sand tiger shark	*Carcharias taurus*	USA	Fin	99.4%	99.3%
Smooth dogfish	*Mustelus canis*	USA	Fin	99.5%	99.7%
Cownose ray	*Rhinopetera bonasus*	USA	Fin	99.6%	99.4%
Scalloped hammerhead (2)	*Sphyrna lewini*	Playita Mia	Muscle	99.8%	100%
Whale shark	*Rhincodon typus*	Galapagos	Muscle	98.0%	97.9%
Bigeye thresher (2)	*Alopias superciliosus*	Santa Rosa	Muscle	100%	98.8%
Pelagic thresher	*Alopias pelagicus*	Santa Rosa	Muscle	99.7%	99.7%
Oceanic whitetip	*Carcharhinus longimanus*	Playita Mia	Muscle	99.7%	97.3%
Shortfin mako (2)	*Isurus oxyrinchus*	Esmeraldas	Muscle	99.0%	99.3%
Long tail stingray	*Hypanus longus*	Santa Rosa	Muscle	98.9%	99.1%
Sicklefin smoothhound	*Mustelus lunulatus*	Santa Rosa	Muscle	99.6%	99.4%
Skipjack tuna (1)	*Katsuwonus pelamis*	Playita Mia	Muscle	99.7%	99.1%
Yellowfin tuna	*Thunnus albacares*	Playita Mia	Muscle	97.4%	98.8%
Skipjack tuna (2)	*Katsuwonus pelamis*	Playita Mia	Muscle	99.4%	98.9%
Tiger shark	*Galeocerdo cuvier*	Santa Rosa	Muscle	99.8%	98.1%
Black tip reef shark (captive)	*Carcharhinus melanopterus*	France	Fin	95.8%	98.0%
Zebra shark (captive)	*Stegastoma fasciatum*	France	Fin	98.2%	97.9%

Note: (1) and (2) indicate where there are multiple samples for the same species; NA = where sequences were inconclusive/sequencing failed.

### 2.2. Confirmation of species identification

DNA was extracted from ~25 mg of tissue from the 26 different species of elasmobranchs and teleost fish (*n* = 31; Table 1) using a Bioline ISOLATE II Genomic DNA Kit (Bioline, UK) according to the manufacturer’s instructions. In the final step of the kit-based extraction, samples were eluted with nuclease-free water (Merck, Germany) in place of elution buffer to ensure compatibility with LAMP. DNA from these 26 species of elasmobranchs and teleost fish were used for validation of species identity for testing species-specific primers in LAMP (section 2.4).

Species identifications were confirmed via PCR and Sanger sequencing. Fish primers FishF1: 5′-TCAACCAACCACAAAGACATTGGCAC-3′ and FishR1: 5′ TAGACTTCTGGGTGGCCAAAGAATCA-3’, were used to amplify ~655 bp of DNA from the COI region of the mitochondrial genome [25]. PCR was performed in a total volume of 20 μL, which included, 10 μL of 2x MyTaq^TM^ Red Mix (Bioline, UK), 0.4 μL of both forward and reverse primers (20 μM), 1 μL of DNA (5 ng/μL), and 8.2 μL of nuclease-free water. All PCR reactions were run with a negative control (no template DNA) and positive control (scalloped hammerhead). Gel electrophoresis prior to sequencing demonstrated a product size of approximately 650 bp in length. ExoSAP-IT™ Express (Thermo Fisher Scientific, UK) was used to treat PCR products prior to sequencing. Briefly, 2 μL of ExoSAP-IT Express reagent was added to 5 μL of PCR product followed by incubation at 37°C for 4 minutes and another incubation period at 80°C for 1 minute.

Approximately 20 ng of DNA for both forward and reverse sequences were sent for Sanger sequencing at the Medical Research Council Protein Phosphorylation and Ubiquitylation Unit (MRC PPU) (Dundee, Scotland) (Table 1). Resulting sequences were confirmed by eye, trimmed for quality (~50 bp from 5’ and 3’ ends) and any residual primer sequences were removed. Forward and reverse sequences were then aligned in BioEdit v7.05, and a Basic Local Alignment Search Tool (BLAST) (https://blast.ncbi.nlm.nih.gov) was used to confirm species by comparing the sequences against all taxa in GenBank.

### 2.3. LOC: Loop Mediated Isothermal Amplification (LAMP)

#### 2.3.1. Primer design

PrimerExplorer v5 (http://primerexplorer.jp/lampv5e/index.html) was used to develop species-specific primers for three CITES-listed species of sharks: bigeye thresher, pelagic thresher and shortfin mako shark. Primers for bigeye thresher shark (KC204935) were developed from the nuclear ITS2 region. Primers for pelagic thresher (KF020876) and shortfin mako shark (MH760159) were developed based on the non-coding mitochondrial D-loop region [42] (Table 2; S1 Table in S1 Appendix). ITS2 and D-loop genes were chosen for primer development because of their high mutation rates and variation between species. ITS2 has been previously used to develop species-specific primers in sharks [28], and therefore was chosen for the bigeye thresher shark. For the full primer development process see Supplementary Information (SI) 1.

**Table 2:** LAMP primers for bigeye thresher shark (*Alopias superciliosus*), pelagic thresher shark (*A*. *pelagicus*) and shortfin mako shark (*Isurus oxyrinchus*).

Species	Primer Sequences (5’-3’)
Bigeye thresher shark	F3: TCCGGATGGTAGCCGTGG B3: GGAAGGAGCCTCAACTCCAG FIP: GGACCAAACCAGTCACTGCGTTCAGGTGCAGGCGTTACC BIP: GCTGGTGGTGTGTTCGCTTTGGGCGTCAGCGCAGCCAA LF: GCTCCGCTTCACCTCCTAC LR: TGGCATTTCGGACGTGAGT
Pelagic thresher shark	F3: ATTTGTGGCACTGCACTC B3: CTCGGTGTCCCAGATCAG FIP: GGTACATTCATTCTTGACGCGATTACTAATCCCCATTAATTGACCAG BIP: CTCCCTTTTATGCCATTTTCGTCCAGTAATTGCTTCATCCCCG LR: TTGATCGTCTCAAGATTTCTTGTCC
Shortfin mako shark	F3: CCCCATTACTGTACTAATCACT B3: GGGATTAATCGAGTACAGCG FIP: GAGGGTGGAAGGAGTAATATGATGATTTCATTACACTCTATTCTTAGTCC BIP: ATCTCTGTATATCTTATGCGGGCTCACAAATAGAGCAATTTTTTCCT LR: GGTAAGAACATCACATCCCGC

LAMP primers consist of a forward primer (F3), forward inner primer, which is made up of F1c and F2 (FIP), a reverse primer (B3), reverse inner primer make up of B1c and F2 (BIP) and optional loop forward (LF) and/or reverse primers (LR). Primers (FIP/BIP/F3/B3) were generated using the default settings in PrimerExplorer, and for each primer sequence a BLAST search against all taxa in GenBank was performed. Loop primers (LF/LB) were developed for each species by inputting the LAMP primers through PrimerExplorer and automatically generating loop primer sets. Loop primer sequences were also run against all taxa in GenBank using BLAST.

#### 2.3.2. Amplification reaction

LAMP was performed in a total volume of 10 μL containing 3 μL of nuclease-free water, 5 μL of WarmStart® Colorimetric LAMP Master Mix (NEB, UK), 1 μL of primers FIP (0.8 μM), BIP (0.8 μM), F3 (0.2 μM), B3 (0.2 μM), LF (0.4 μM) and specific primers for the three sharks (bigeye thresher, pelagic thresher and shortfin mako shark; 2) or Lambda (λ) LF and/or LR (0.4 μM), and 1 μL of DNA (approximately 5 ng/μL). Negative controls, containing no template DNA, were also prepared. LAMP primers for the positive control using λ DNA were obtained from Merck (Germany), and λ DNA was purchased from New England Biolabs (NEB, UK) (250 ng λ DNA, stored in 10 mM Tris HCl pH 7.5, 10 mM NaCl and 1 mM EDTA). For the positive control LAMP reaction, 5 ng/μL of λ DNA was used (more detail on controls can be found in SI 2).

The mixture was incubated at 65°C for 1 hour and then visualised on a 1.5% agarose gel, following electrophoresis, using a Biorad Gel Doc EQ system w/ Universal Hood II (UV transilluminator) (Bio-Rad Laboratories, USA). Successful amplification was indicated by a colour change (pink to yellow) and presence of bands on the gel.

#### 2.3.3. Primer specificity

To ensure amplification of the three target species of sharks, bigeye thresher, pelagic thresher, and shortfin mako shark, the species-specific primers (Table 2) were initially tested against DNA from target species (in triplicate) to confirm successful amplification. The specificity of the primers was then determined by LAMP amplification of 26 different species of elasmobranchs and fish previously confirmed by Sanger sequencing (Section 2.2 and Table 1). A DNA concentration of approximately 5 ng/μL was used for all subsequent reactions. All reactions were performed in triplicate for each primer set, and amplification was further confirmed by gel electrophoresis.

### 2.4. LOC and LAMP optimisation

#### 2.4.1. LOC design & fabrication

**Fig 1:**
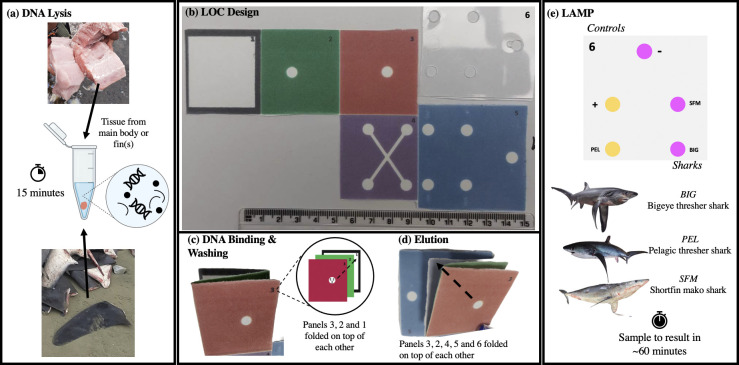
a) Overview of the procedure for cell lysis from a small piece of meat or wet fin sample taken from markets; b) Photograph showing the paper LOC design with five different coloured areas (panels) that was folded in a origami-style manner to enable different steps of the genetic analysis to be performed; c) Schematic showing the folding of the LOC for DNA binding and washing steps; d) Schematic showing the alternative folding of the LOC for DNA elution; e) Location of the five LAMP chambers for species-specific amplification, as well as positive and negative controls.

The LOC was designed to integrate DNA extraction, amplification and visualisation using LAMP. Method development and optimisation, including portable lysis and extraction for the LOC can be found in SI 3. The LOC incorporated three CITES-listed sharks namely bigeye thresher, pelagic thresher shark, and shortfin mako shark, as well as a negative and positive control using λ primers and DNA (DNA only for positive control). LOCs were created in Microsoft Word, and a wax design was printed onto Whatman Grade 1 filter paper (Fisher Scientific, UK) using a Xerox ColorQube 8580 Printer (Xerox, USA) to produce the design in Fig 1B. The printed template was then incubated at 130°C for 3 minutes allowing the wax to melt. One Whatman^***©***^ GC/F glass microfiber filters (6.4 mm in size; grade 1.2 μm) (Merck, Germany) was placed in-between panel 2 (green) and panel 3 (orange) on the chip (panel 2 and 3 in Fig 1B and 1C), and five were placed on a separate plastic mould (panel 6 in Fig 1E).

#### 2.4.2. LOC Standard Operating Procedure (SOP)

For lysis, approximately 25 mg of tissue from each of the above species (one species per chip) were added to 500 μL of 5 M GuHCl and agitated using a pipette tip (“cutting”; mechanical lysis) (Fig 1A). The DNA solution was then left at room temperature for 15 minutes. Whilst the sample was lysing, a mastermix was made for each of the sharks, the negative and positive λ control (enough for two reactions), which contained 2 μL of species-specific primer, 10 μL of WarmStart® Colorimetric LAMP Master Mix (NEB, UK), and 8 μL of nuclease-free water for the negative control. Each solution was gently pipetted up and down to mix all the components and left on ice until used on the LOC.

Then, 30 μL of the lysed DNA solution was loaded onto panel 3 (orange) (Fig 1C). Next, 30 μL of 70% ethanol was loaded onto the chip (panel 3 again), and once that was absorbed another 30 μL was added. When the ethanol had dried completely (after ~30 seconds), the waste panel 1 (black) was discarded, and panels 3 (orange) and 2 (green) (in this order) were placed over panels 4 (purple) and 5 (blue) (Fig 1D). Whilst the ethanol was drying, 3 μL of λ DNA was added to the positive control mastermix and mixed by pipetting gently up and down.

The LOC was then re-folded and placed on top of the plastic mould that is stuck onto an adhesive Polyester PCR Sealing Film (Starlab, UK) containing the five glass microfibre filters. Nuclease-free water (100 μL) was gently loaded onto panel 3 (pink) to elute the DNA, which travelled along the channels on the cross-shaped panel 4 (purple) and into the four separate DNA amplification chambers on panel 5 (blue) that sits on top of the plastic mould (panel 6 in Fig 1E). Once the water had been fully absorbed by all four chambers, all paper panels were removed. Next, the LAMP mastermixes were loaded onto the corresponding chambers (6 μL of either bigeye thresher, pelagic thresher, shortfin mako shark chambers, 7 μL for the λ positive control and 10 μL for the negative control).

The plastic LOC was sealed together by folding the adhesive Polyester PCR Sealing Film. The LOC was then then placed onto a portable Miniature Incubator (TC-MIW) with a Temperature Controller (TC-1-100-1) (BioScience Tools, USA) for 30 minutes at 65°C. Each of the chambers on the plastic mould contained the LAMP mix, which turned from pink to yellow if the target species was present. A positive control was also included on the LOC to ensure that the set-up was working, i.e. if the positive control changed from pink to yellow, and the negative control stayed pink (see S1 Fig in S1 Appendix for decision making flowchart). Colour changes of the three sharks was dependent on what species were used for the initial lysis step.

LOCs were tested in the lab on tissue belonging to each of the target species (bigeye thresher, pelagic thresher and shortfin mako shark) as well as one non-target species (blue shark, *Prionace glauca*). Blue shark was chosen as the non-target species as it is commonly found at the fish markets in Ecuador and is an important commercial species globally [19,26]. The LOCs were tested ≥ 3 times per species, pictures were taken before and after to show the colour change from pink to yellow. Positive and negative controls were run on a thermocycler conjunctively to every two LOC’s using the same mastermix with λ DNA (positive control only), nuclease-free water and λ primers.

#### 2.4.3. Testing of real-world samples and workshop

A small-scale preliminary test was carried out in Ecuador in 2022 on six LOCs namely two bigeye threshers, two pelagic threshers, and two shortfin mako sharks. These samples were collected opportunistically from markets in Santa Rosa and Manta, Ecuador.

A two-day workshop, held in August 2023, about the LOC devices was conducted in Manta, Ecuador with 31 attendees from various departments across the Viceministerio de Acuacultura y Pesca. The workshop consisted of: i) a lecture and practical guide to shark identification using genetic analysis techniques; ii) hands-on demonstration of the LOC devices; and iii) participant feedback. Participants completed a pre- and post-workshop survey, including Likert scale questions to assess participants’ knowledge and experience (1 = nothing, 2 = very little, 3 = more or less, 4 = very good and, 5 = excellent), as well as more open-ended questions to allow participants to provide constructive feedback on the LOC devices. These findings were used to further optimise the LOC. Ethical approval was granted by the Faculty of Science and Engineering Research Ethics and Governance Committee at Manchester Metropolitan University (application number 7521). All workshop participants provided written consent.

## 3. Results and discussion

### 3.1. Lab-on-a-Chip (LOC): Lysis and extraction

A LOC was successfully designed to integrate field-based DNA extraction, amplification and visualisation using LAMP into a single cost-effective system, which could be employed by non-specialists to monitor the trade in sharks and shark products.

#### 3.1.1. Optimisation and evaluation of cell lysis techniques

Although molecular techniques are advancing rapidly and we are now able to amplify and sequence DNA in the field (e.g., MinION), there has been limited research on portable extraction techniques, including cell lysis [43]. We have applied a lysis method that incorporates easy steps that can be carried out in the field by combining the use of a chaotropic salt (5 M GuHCl) with mechanical disruption in a single test tube (S2 and S3 Figs in S1 Appendix). This removed the need for common laboratory equipment (e.g., vortex, incubators, and centrifuges) and numerous steps involving different chemicals (e.g., proteinase K, lysis buffer 1 and 2). Despite the simpler, portable nature of the lysis method, it was still effective on complex samples, such as shark fin, which are made of cartilage with very little muscle tissue and a lot of collagen fibres making them rigid and tough [44].

#### 3.1.2. Optimisation and evaluation of field-based DNA extraction

**Fig 2:**
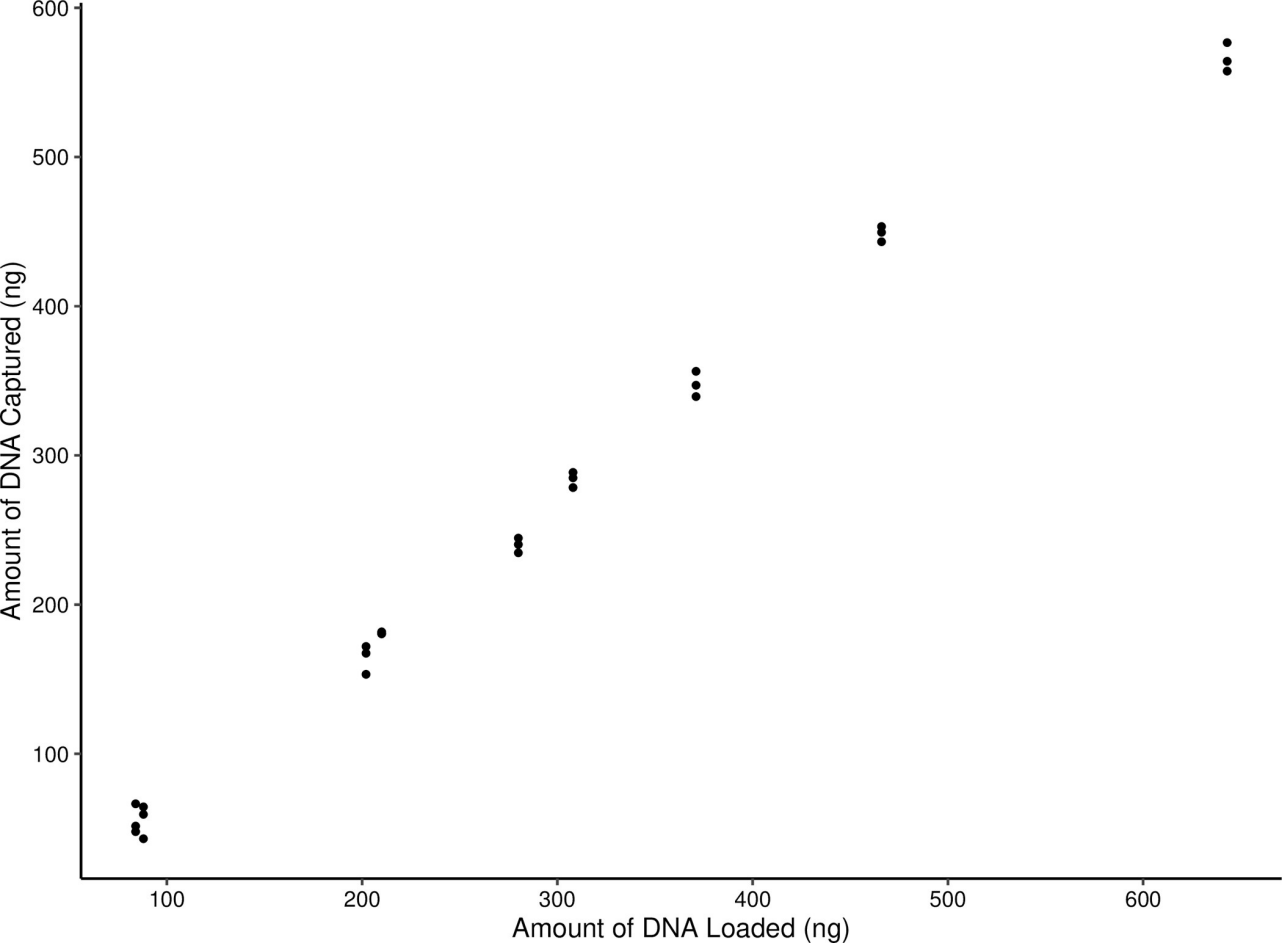
DNA capture efficiency of Whatman© glass microfiber filters (GF/C). The DNA solution was made up to 25 μL with 5M GuHCl in concentrations ranging from 8 to 64 ng/μL. The maximum retention of the GF/C filters were recorded.

We aimed to develop a portable extraction method that could produce high yields of DNA whilst also being simple, cost-effective, and rapid. The lysis method above using 5 M GuHCl was used to determine the capture efficiency of the GF/C filters on the LOC. The GF/C filter used per reaction had an average capture efficiency of 83.6%, and the total amount that could be bound was 260 ng of DNA, which is more than adequate for downstream application as LAMP only requires as little as 6 copies of DNA for successful amplification (Fig 2) [45]. The GF/C filters provide a straightforward and cost-effective method of capturing DNA on the LOC and offer versatility as the user can cut them into whatever shape or size necessary for DNA capture [46].

**Fig 3:**
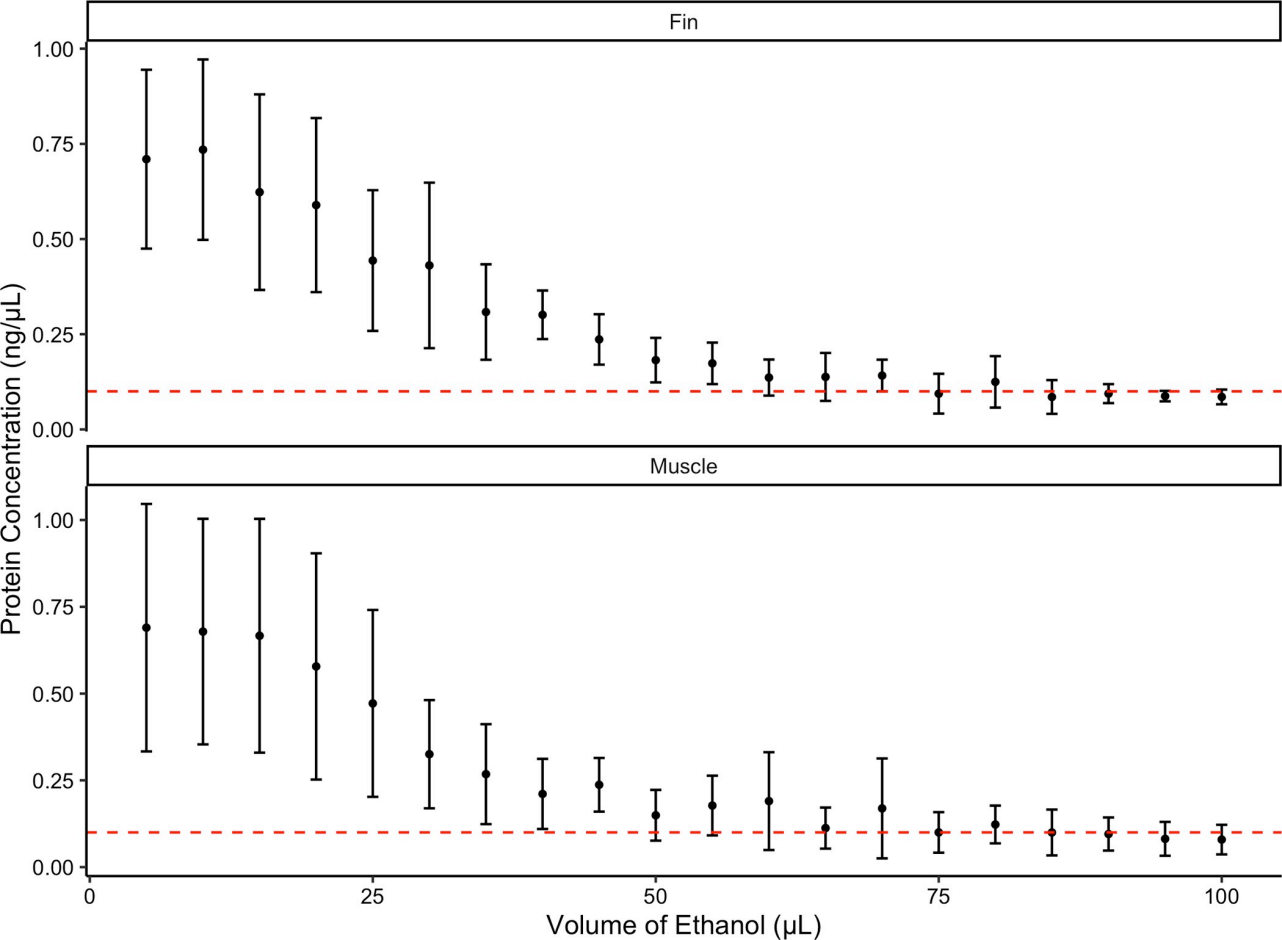
DNA from fin and muscle tissue samples was purified using 70% ethanol in 5 μL increments. The protein concentrations of the resulting samples were compared. The red dashed line indicates the plateau in protein concentration at 0.1 ng/μL when 60 μL of ethanol is loaded onto the LOC.

Once bound, it was important to then wash the DNA to remove any impurities, which may inhibit the subsequent LAMP reaction, and therefore a purity (A_260_/A_280_ nm ratio) ranging between 1.7 and 2.0 [47] was required. The volume of ethanol loaded onto the LOC depended on tissue type, impurity levels and protein concentration. The optimal volume of ethanol required to remove most impurities from both fin and muscle tissue samples was 60 μL (Fig 3). At 70 μL the GF/C filter became oversaturated and the LOC could not maintain its structural integrity. Subsequently, 100 μL of nuclease-free water were then needed to physically transfer the eluted DNA into the amplification chambers (Fig 1D & 1E).

### 3.2. LAMP

#### 3.2.1. Primer specificity

The specificity of the LAMP primers was tested against the target species, as well as 26 non-target species as listed in Table 1. LAMP primers for all three sharks were specific, only amplifying DNA from the target species (see S4–S8 Figs in S1 Appendix for colour change and gel electrophoresis results). The primers proved to be specific even when tested against closely related species such as the common thresher shark (A. vulpinus), great white shark (Carcharodon carcharias) and salmon shark (Lamna ditropis) which are in the same Order as the three target sharks, and the common thresher shark in the same genus as bigeye and pelagic thresher shark (Alopias spp.) [48,49].

Species identification is predominantly carried out using DNA barcodes that amplify specific regions of DNA (e.g COI, cytochrome B, NADH2) or limited nuclear genes (e.g., ITS2) between species. The LAMP primers developed here are designed to amplify only one species. Developing completely species-specific LAMP primers for additional species of sharks may prove challenging as DNA is highly conserved and mutation occurs at very slow rates in sharks [50], however, we have successfully demonstrated this for t bigeye and pelagic thresher sharks which are very closely related to each other and to other sharks within their order (Lamniformes) [48,49].

### 3.3. Integrated LOC

#### 3.3.1. LOC Standard Operating Procedure (SOP)

**Fig 4:**
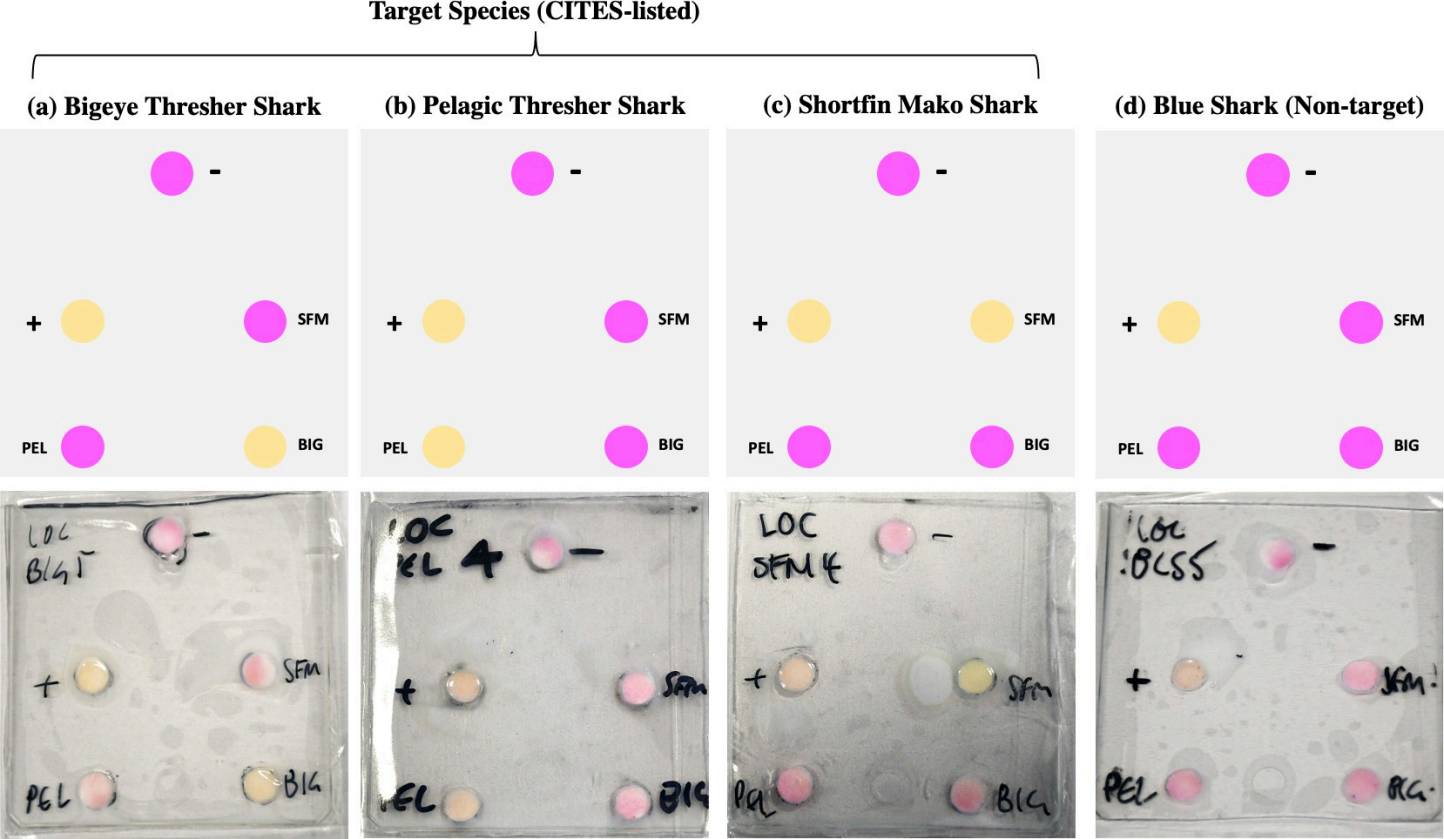
Schematic (*top panel*) and photographic (*bottom panel*) examples of LOC results showing amplification of target species (a) bigeye thresher shark (*Alopias superciliosus*), (b) pelagic thresher shark (*A*. *pelagicus*), and (c) shortfin mako shark (*Isurus oxyrinchus*), and no amplification of non-target species (d) blue shark (*Prionace glauca*).

The LOC uses a color change from pink to yellow when DNA amplification of a target species takes place, allowing for a simple “yes” or “no” answer. The optimised lysis, extraction, and amplification (using LAMP) were combined on a LOC, which were then tested against the three target species of sharks (bigeye thresher, pelagic thresher, and shortfin mako shark) and one non-target species of shark (blue shark) in the lab (n ≥ 3). Results indicated successful amplification of each target species, as well as amplification of the positive control and no amplification of the negative control. Example of the LOC results are shown in Fig 4 (full details can be found in S9 Fig in S1 Appendix). There were no instances of cross-contamination for the non-target blue shark (n = 4). For bigeye thresher and shortfin mako shark, one false negative was observed for each (S10 Fig in S1 Appendix).

For our method development, we incorporated the use of a portable miniaturised incubator (~$1,500) for reliable results but for future applications of the LOC in the field any heating or incubating device (e.g., heating plate or hot water bath) could be used that can supply a constant temperature of 65°C for 30 to 60 mins. This operating system is much simpler than that required for PCR, which relies on complex temperature control for stages of denaturation, annealing and extension of DNA/RNA sequences. Although there are currently many methods of identifying shark products, for example visually using fin and meat guides [51,52], 3D fins with TRAFFIC [53], mini-DNA and DNA barcoding [26,27,54], many of these techniques are costly, rely on specialist for visual identification of whole caught sharks or fins, laboratory facilities and experts to carry out genetic analyses, or are time-consuming.

Although the current focus of this study was on the development and application of the LOC for the identification of CITES-listed shark species, the LOC’s versatility allows adaptation to include other elasmobranch species or taxa by optimising cell lysis conditions for different sample types and changing LAMP primers. This broadens the utility of the LOC and indicates its potential for including other species in the illegal wildlife trade, for example by encompassing elephants (ivory), rhino horns, tiger bones and fish maw, as well as to other areas such as ecological or life-history studies.

#### 3.3.2. Testing of real-world samples and workshop

LOCs were initially tested in the field to identify shark species from six fresh muscle tissues of sharks landed at commercial fish markets in Ecuador. The LOCs were tested without the use of a laboratory or any laboratory equipment in a hotel room. Of the six LOCs, five worked successfully (one bigeye thresher shark and four shortfin mako sharks) and one failed due to a false negative. This demonstrates proof-of-concept in the field but would require an increased number of tests to be carried out for validation, particularly to demonstrate usability in remote areas. The key learnings from this field-study were how to store the LAMP mix during long-haul flights (>14 hrs) and in the field to ensure no CO_***2***_ entered the vials, importance of the distance of the negative control chamber from the sample chamber, and drying time needed for the removal of contaminants step on the LOC (using 70% ethanol).

The LOC works as a screening test and therefore the cost per sample is less compared to PCR, $6.00 vs. $13.3 prior to Sanger sequencing which will further increase the cost (for further breakdown of cost see S3 Table in ***S1 Appendix***). As the LOC is made from paper and uses fewer reagents, the ecological footprint is also significantly reduced. The LOC is also rapid (less than an hour from extraction to visualisation), field-based and can be carried out by non-scientifically trained personnel. It is important to note that non-scientifically trained personnel require some initial training prior to using of the LOC. Consideration would also need to be given to ensure that the correct LOC was deployed in a given study area, which would require prior knowledge of which species are present and therefore initial input from a trained specialist.

As the LOC works as a screening test, further laboratory analyses would be required to determine species identity to an accredited standard, for example relating to the handling of illegal products (e.g., dried fins from CITES-listed sharks). The LOC can reduce the number of samples that would further require downstream laboratory analyses and greatly reduces the cost as only a few samples need to be sequenced rather than all unknown samples. This is especially true in the case of high volumes of unidentified shark fins or unlabelled meat which can be expensive, approximately $10 per sample. In 2019, one of the largest seizures was recorded to date in the Galápagos Marine Reserve, where an illegal shipping vessel contained over 7,600 sharks [55]. The sample cost can be reduced to $0.94 and can be done in <4 hours using a multiplex real-time PCR assay [54], but this still requires a Real-Time PCR machine which may not be present in every lab and is costly.

Collaborating with local stakeholders and communities is vital in conservation efforts. We carried out a workshop with valuable stakeholders in the initial stages of developing the LOC to gain valuable feedback on its application in Ecuador. Ministerial officials (*n* = 31) were shown some genetic techniques and a demonstration on the use of the LOC. Of the 31 ministerial officials who attended the LOC workshop, 18 had experience of carrying out species identification, with 22% having experience in visual identification, 22% in use of the genetic techniques and 38% with both. Over 80% of the participants had either worked with or confiscated shark products as part of their roles. Following the workshop, the participants’ knowledge of LOC technology increased from average of 1.88 to 3.80. All participants (100%) responded positively that the LOC would be useful, highlighting benefits for identification of CITES-listed species in processed products where visual identification would not be possible and to verify exports at border control. Constructive feedback revolved around inclusion of additional CITES-listed species but not just restricted to sharks, e.g., mobula rays (Mobulidae), and further development on the SOP for the LOC.

## 4. Conclusion

We present the first completely field-based technique in the form of a paper-based LOC that can used to identify threatened and CITES-listed species of sharks. Previous LOCs incorporate one or two stages of DNA extraction, amplification, and visualisation; we provide all three. LOC technology is still an up-and-coming field. Despite the 15 years of research, most of their applications have been on clinical diagnostics and biomedical research; there has been limited to no research on the use of LOC devices for conservation. There is still a great deal of uncertainty surrounding the techniques used, and therefore our LOC for identifying sharks is a proof-of-concept and can provide a screening of shark species detected but further validation is required. Whilst this work was carried out in Ecuador, the LOC can be applied to any market globally and further development could see the inclusion of other CITES-listed elasmobranchs or taxa entirely. The LOC provides us with the ability to identify sharks in the field without the use of expensive laboratory equipment and can distinguish between sharks, rays and fish, and identify the three CITES-listed sharks: bigeye thresher, pelagic thresher and shortfin mako shark.

Despite recent genetic advances in identifying sharks, there is still an urgent need to involve local stakeholders in the conservation of sharks. Attendees of the workshop reported that the LOC would be useful, in identifying processed products or at border control. Genetic tools should be available to non-specialists and people with limited access to expensive or specialised equipment, especially in countries where sharks are targeted the most, and where there are fewer regulations on the sale of shark and shark-related products.

## Supporting information

S1 Appendix (DOCX)
