# Supplementary material for: Genetic identification of three CITES-listed sharks using a paper-based Lab-on-a-Chip (LOC)
Source: PLoS One. 2024 Apr 4;19(4):e0300383. doi: 10.1371/journal.pone.0300383 (PMC10994358; doi:10.1371/journal.pone.0300383)
Supplement: S1 Appendix — (DOCX) [file pone.0300383.s001.docx]

Supplementary Information (SI): Genetic identification of three CITES-listed sharks using a paper-based Lab-on-a-Chip (LOC)

**SI 1: Primer Design**

Table S1: Sequences used for LAMP primer development.

| **Common Name** | **Species** | **Type of Sequence** | **Gene** | **Genbank Accession No.** | **Length (bp)** |
| --- | --- | --- | --- | --- | --- |
| Biegeye thresher shark | Alopias superciliosus | Nuclear ribosomal RNA (rRNA) | ITS2 locus (located between 5.8S and 28S) | KC204935 | 740 |
| Pelagic thresher shark | Alopias pelagicus | Mitochondrial DNA (mtDNA) | D-loop (1563 – 16692 bp) | KF020876 | 1059 |
| Shortfin mako shark | Isurus oxyrinchus | Mitochondrial DNA (mtDNA) | D-loop | MH760159 | 791 |

Using the sequences above, primers were developed in Primer Explorer V5 (<http://primerexplorer.jp/lampv5e/index.html>) for the three CITES-listed sharks: bigeye thresher, pelagic thresher and shortfin mako shark. Outlined here is a full overview of how the primers were developed (using bigeye thresher shark as an example).


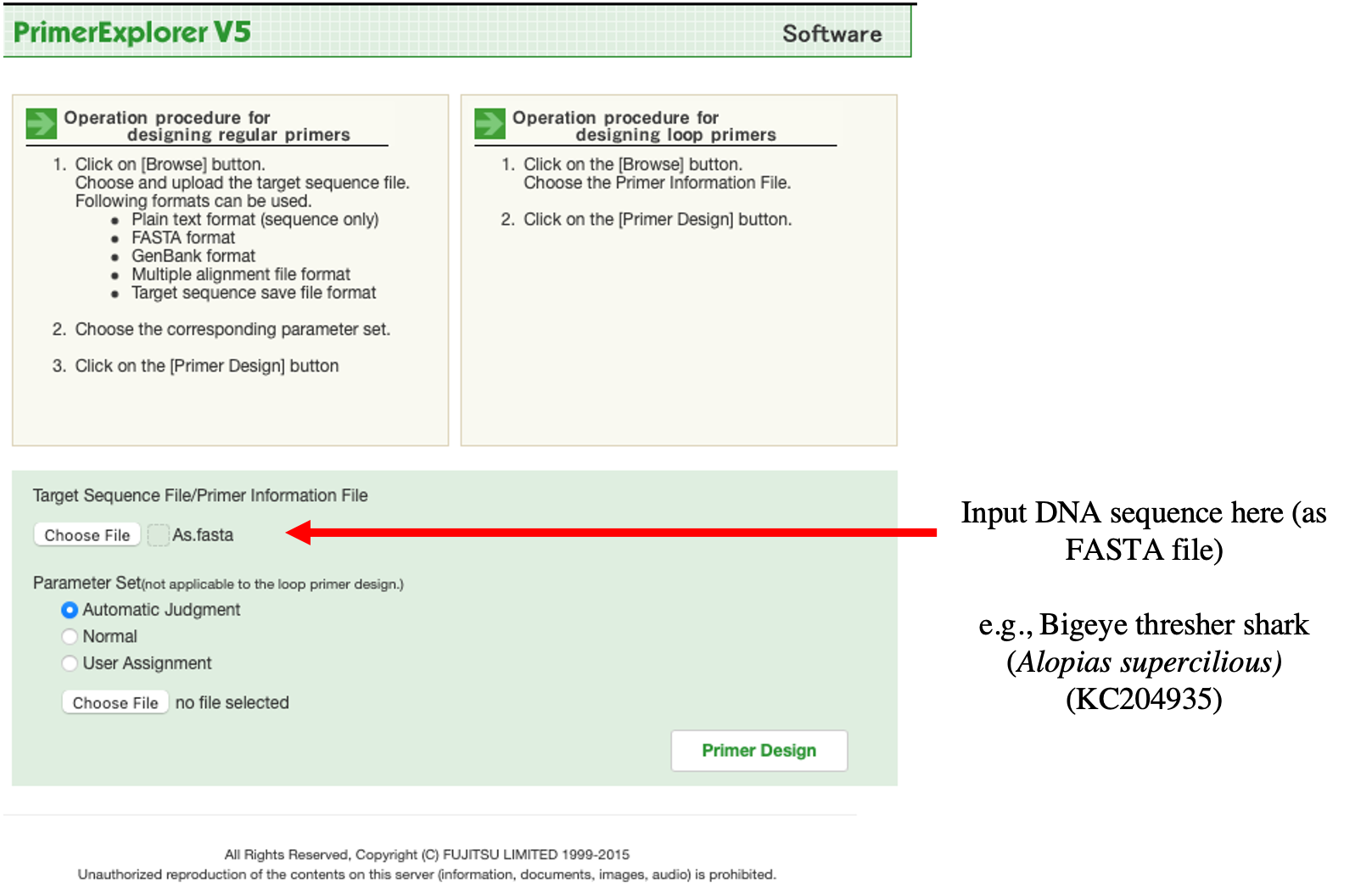


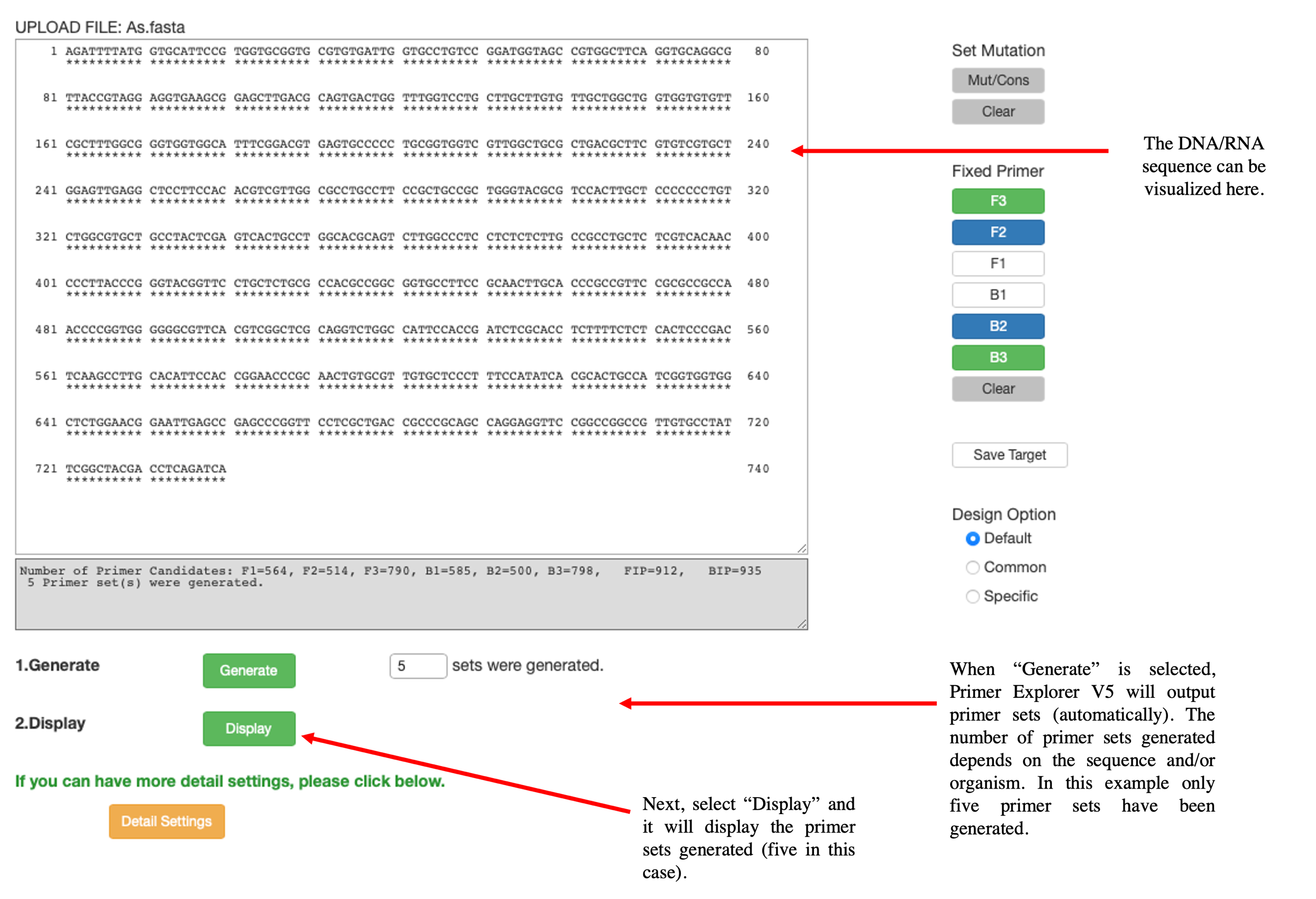


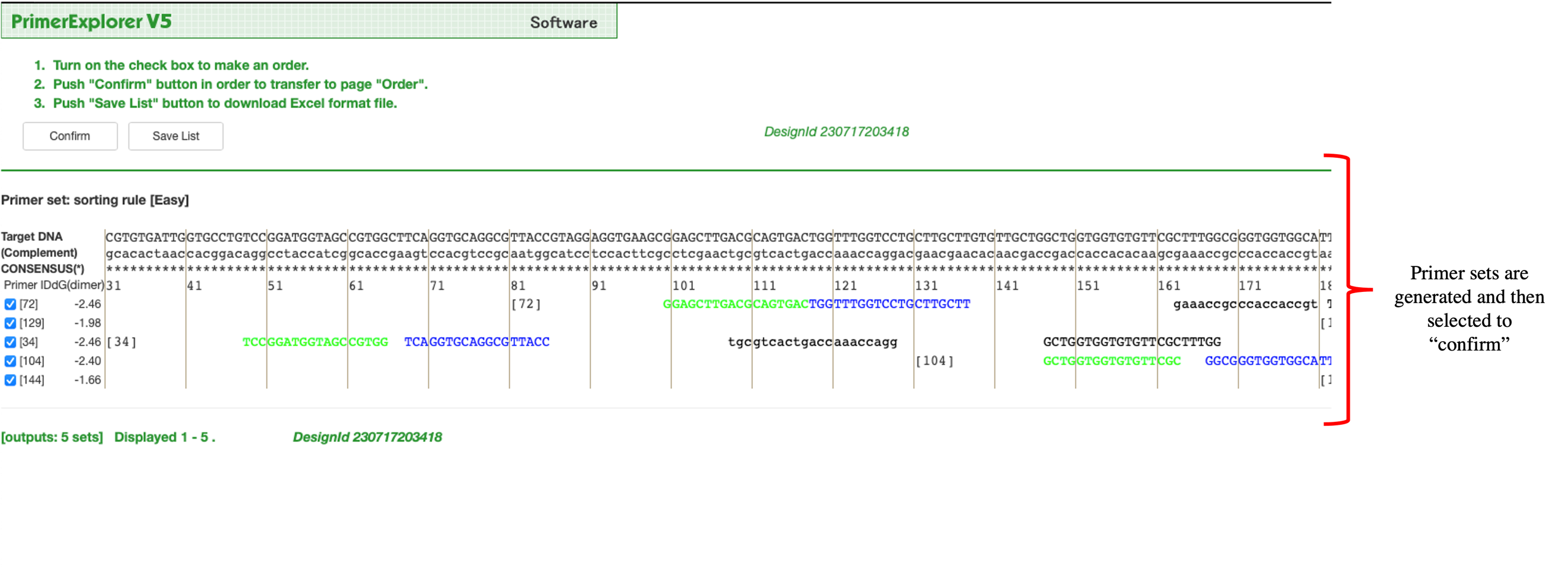


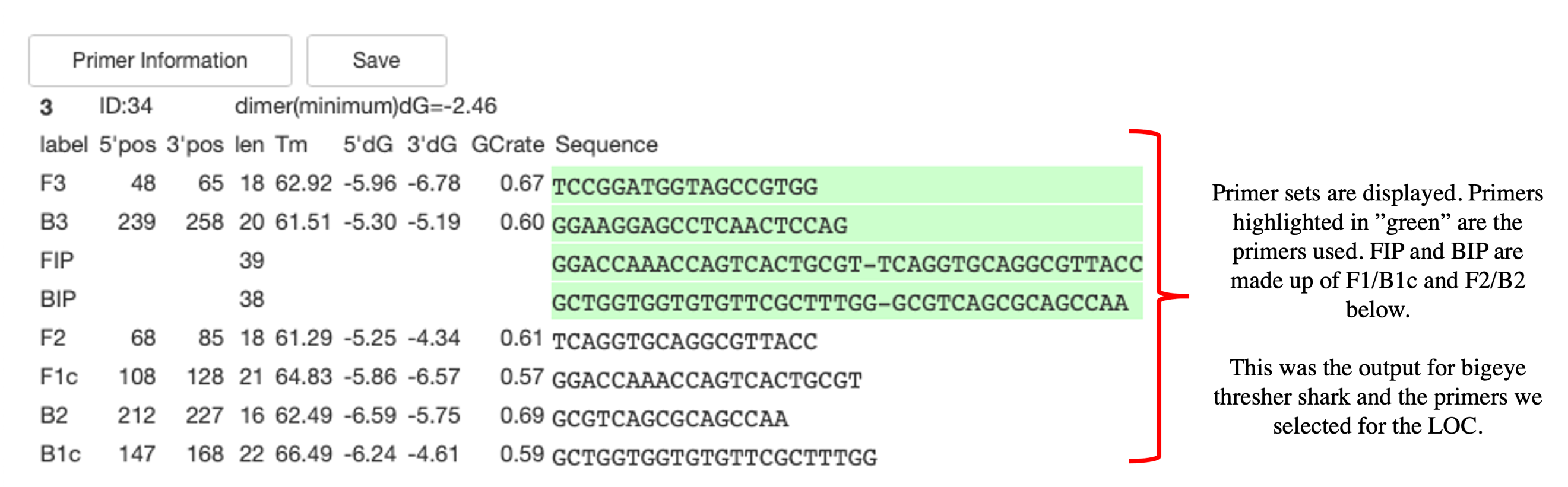


The next step is to BLAST each of the primer sets generated against “all taxa” in BLAST (<https://blast.ncbi.nlm.nih.gov/Blast.cgi>) with the aim to produce a high match with your target species, and lower match with non-target species. In our case, we were aiming to have a lower match with other elasmobranchs and teleost fish. Then once a suitable primer group are found, they are put back into Primer Explorer V5 to create loop primers.

**SI 2: Controls**

A positive control was incorporated onto the LOC (Figure 1; main manuscript) using λ primers (Merck, Germany) and readily available λ DNA (NEB, UK). The design of the LOC physically separated the negative control from the other chambers (including target species primers and positive control) by less than 1.33 cm to ensure that it was DNA free. For the positive control, 5 ng/µL of λ DNA was required to produce a consistent reaction, any lower and false negative were observed. The positive and negative controls (including primers and DNA) were added to the LOC after elution of DNA. A mastermix was made for the positive and negative containing 10 µL of LAMP mix and 2 µL of λ primers. For the negative control an additional 8 µL of nuclease-free water (Merck, Germany) was added, and for the positive control, 2 µL of 5 ng/µL λ DNA (NEB, UK) was added. No water was added to the positive control as this was added during elution of DNA on the LOC. Both mastermixes were homogenised by pipetting up and down gently until the solution was fully mixed. Seven µL of the positive control mastermix was pre-loaded onto the positive control chamber, and 10 µL of negative control mastermix in the negative control chamber on a separate plastic chip (mould with five chambers containing each a Whatman© glass microfiber GF/C filter (Merck, Germany). For the negative control, the distance from the reaction wells was important. Any detectable failure in the controls meant that the LOC was discarded immediately (see Figure S1 for decision making flowchart).

**
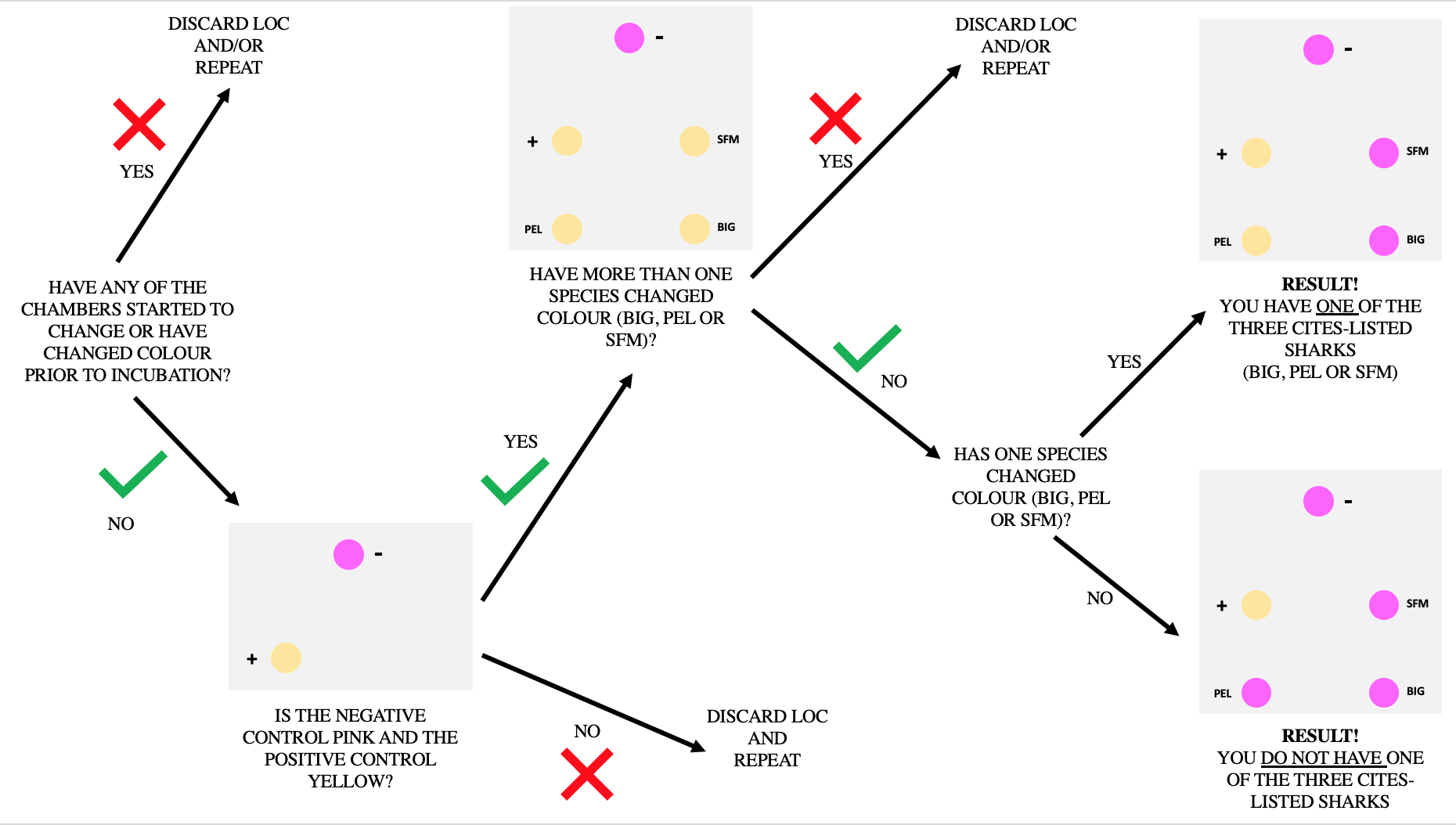
**

Figure S1 Decision making flowchart for the LOC Standard Operating Procedure (SOP) where BIG = bigeye thresher shark, PEL = pelagic thresher shark and SFM = shortfin mako shark. The (+) and (-) indicate positive and negative control. The positive should always turn yellow and negative stay pink.

**SI 3: Method Development & Optimisation**

**Comparison of Lysis Methods**

For optimisation experiments, a variety of cell lysis methods were evaluated using ~25 mg of either fin or muscle tissue belonging to scalloped hammerhead (*Sphyrna lewini*). Methods included thermal lysis, chemical lysis, chemical lysis with the addition of surfactants and mechanical lysis. Methods were compared to find a field-based lysis method that gave comparable results to conventional DNA extraction kit, with consideration given to speed, amount of DNA yielded, quality of DNA, cost of analysis and ease of use. DNA from the scalloped hammerhead was used as the model species for all the testing of LOC processes in sections 2.3.1. – 2.3.3 (main manuscript) as good quality tissue was readily available.

For the lysis of tissue using a DNA extraction kit, tissue samples were added to 180 μL of Lysis Buffer GL and 25 μL Proteinase K solution following the pre-lysis steps of the Bioline ISOLATE II Genomic DNA Kit (Bioline, UK) protocol. Samples were then incubated at 56°C for 3 hours until completely digested Samples were vortexed briefly and added to 200 μL Lysis Buffer G3, and incubated at 70°C for 10 min. For thermal lysis, tissue samples were added to 500 µL nuclease-free water and incubated at 100 °C for 15 minutes.

For chemical lysis, tissue samples were added to 500 µL guanidine hydrochloride (GuHCl) (Thermo Fisher Scientific, UK) of varying concentrations ranging from 3M to 8M [1-6]. Samples were left at room temperature for 5, 10 and 15 minutes.

Mechanical lysis was carried out in 5M GuHCl with 2% SDS, different mechanical lysis techniques were used including no disruption (control), cutting, pipetting, rolling, shearing, and vortexing (lab equipment) (Table S2). Vortexing was carried out using a vortex mixer found in the laboratory. It was used as to compare DNA concentrations yielded from laboratory equipment versus “field-based” mechanical lysis methods.

The total DNA concentration (ng/µL) and purity (260:280 nm ratio) of the lysed DNA samples were analysed using a Thermo Scientific™ NanoDrop 2000 spectrophotometer (Thermo Fisher Scientific, UK).

Table S2: Mechanical lysis methods used to physically break down cell membranes.

| **Method** | **Description** |
| --- | --- |
| Control | The samples were not disturbed. |
| Mashing | A pipette tip was used to break apart fin and tissue samples for 15 seconds. |
| Pipetting | Samples were pipetted up and down ten times using a 1000 µL pipette set at 500 µL. |
| Rolling | Samples in Eppendorf tubes were rolled across a tube rack for 15 seconds. |
| Shearing | Samples were sheared up and down 15 times with a needle syringe. |
| Vortexing | Samples were agitated for 15 seconds using a vortex (laboratory equipment). |

Statistical analysis and data visualisation were carried out in R 3.6.1 [7]. Data was tested for normality with all variables found to be not normally distributed; non-parametric statistical methods were used. Wilcoxon rank-sum tests were used to compare differences in DNA concentrations between fin and muscle tissue. Kruskal-Wallis rank-sum tests and pairwise multiple comparison test (‘Kruskalmc’) using the “pgirmess” package [8] were used to assess differences in DNA concentrations across different lysis methods, including chemical and mechanical lysis.

The lysis methods were tested to establish the most suitable method for field-testing that were cost effective and efficient. When evaluating chemical lysis methods, significant differences were observed between GuHCl concentrations used and total DNA released for fin (Kruskal-Wallis: *χ*^2^ = 14.344, df = 5, p < 0.02; 78.7±38.3 ng/μL, Figure S2) and muscle tissue (Kruskal-Wallis: *χ*^2^ = 16.013, df = 5, p < 0.01; 39.3 ± 28.0 ng/μL, Figure S2). Five molar GuHCl was chosen as the optimum concentration as it yielded the highest concentrations of DNA from muscle and fin tissue (90.6 ± 8.22 ng/μL and 113.4 ± 18.8 ng/μL, respectively). Using the same concentration of GuHCl for both samples also increased the simplicity for end users as the lysis method is consistent and therefore not dependant on what type of sample is used (i.e., whether it is fin or muscle tissue).

Significant differences in total DNA concentration were observed between the mechanical lysis methods for fin (Kruskal – Wallis: *χ*^2^ = 16.112, df =8, p < 0.05, Figure S3) and muscle tissue (Kruskal – Wallis: *χ*^2^ =18.5, df = 8, p < 0.02, Figure S3). Pipetting yielded higher concentrations of DNA in fin tissue (374 ± 19.7 ng/µL) and cutting (386 ± 102 ng/µL) in muscle tissue, though the multiple comparison test revealed no significant differences (Figure S3). Mechanical disruption by mashing was able to successfully yield high concentrations from both fin and muscle tissue.

**Optimisation of Field-based DNA Extraction Protocol**

Scalloped hammerhead DNA was lysed in 5 M GuHCl and used to determine the retention (capture efficiency) of the Whatman^©^ glass microfiber filters (GF/C) (Merck, Germany) (Figure 2; main manuscript). DNA concentrations (ng/µL) for all subsequent reactions were quantified using NanoDrop 2000 spectrophotometer.

The lysed DNA was made up to 25 µL with 5M GuHCl in concentrations of DNA ranging from 8 to 64 ng/µL and loaded onto the Whatman^©^ GF/C glass microfiber filters to determine their binding efficiency. The maximum retention of the GF/C filters were recorded by collecting the DNA solution in a 0.2 mL PCR tube and recorded in the DNA concentration (ng/µL) for each eluate.

Next the bound DNA samples underwent a wash step to remove any contaminants. A 70% ethanol solution was loaded onto the LOC in 5 µL increments until a total of 100 µL had passed through the GF/C filter. Eluted fractions were collected on the other side of the LOC in 0.2 mL PCR tubes, and the protein and DNA concentrations (ng/µL) as well as purity were recorded. DNA was then eluted using 100 µL of nuclease-free water.


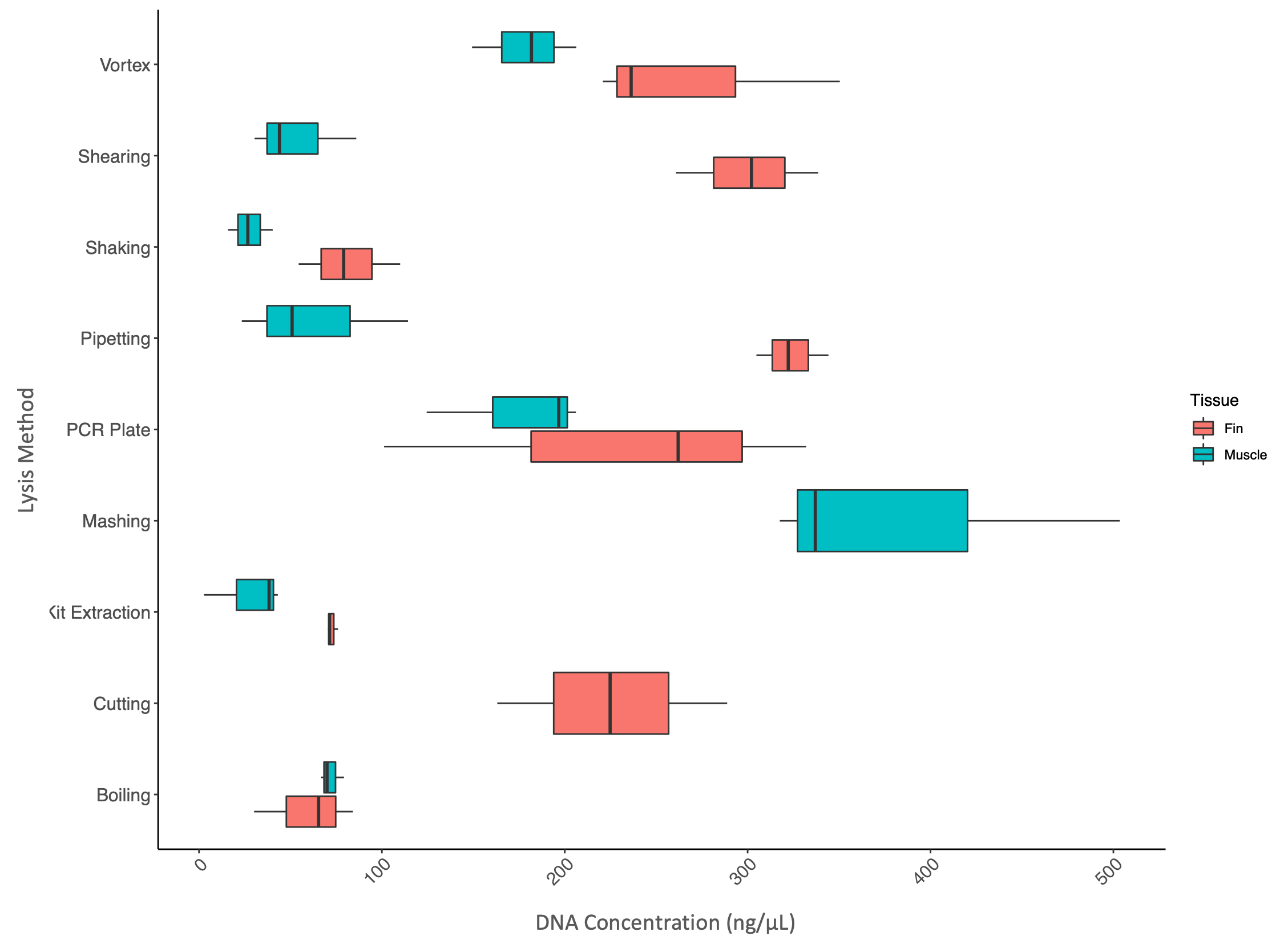


Figure S2 Comparison of different mechanical lysis methods used to yield DNA from fin and muscle tissue of scalloped hammerhead sharks (*Sphyrna lewini*).


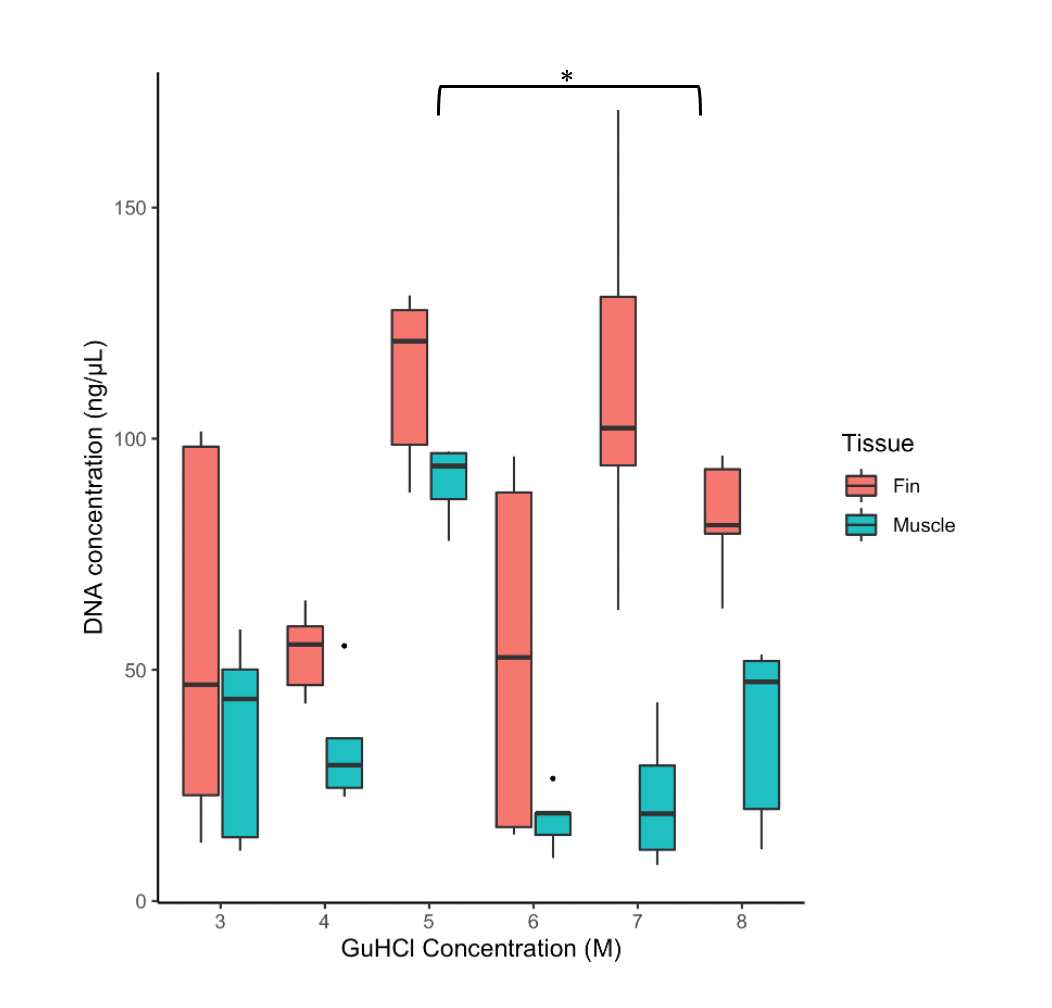


Figure S3 Comparison of different concentrations of GuHCl (ranging from 3 M to 8 M) used to obtain DNA from 25 mg of fin and muscle tissue of scalloped hammerhead sharks (*Sphyrna lewini)*.

**Primer Specificity**

All three primer sets (bigeye thresher (Figure S4), pelagic thresher (Figures S5&S6) and shortfin mako (Figure S7&S8) shark proved to be specific when testing against the 26 elasmobranch and teleost fish species (Table 1). Repeat assays were carried out where contamination was present and revealed these were caused by human error.


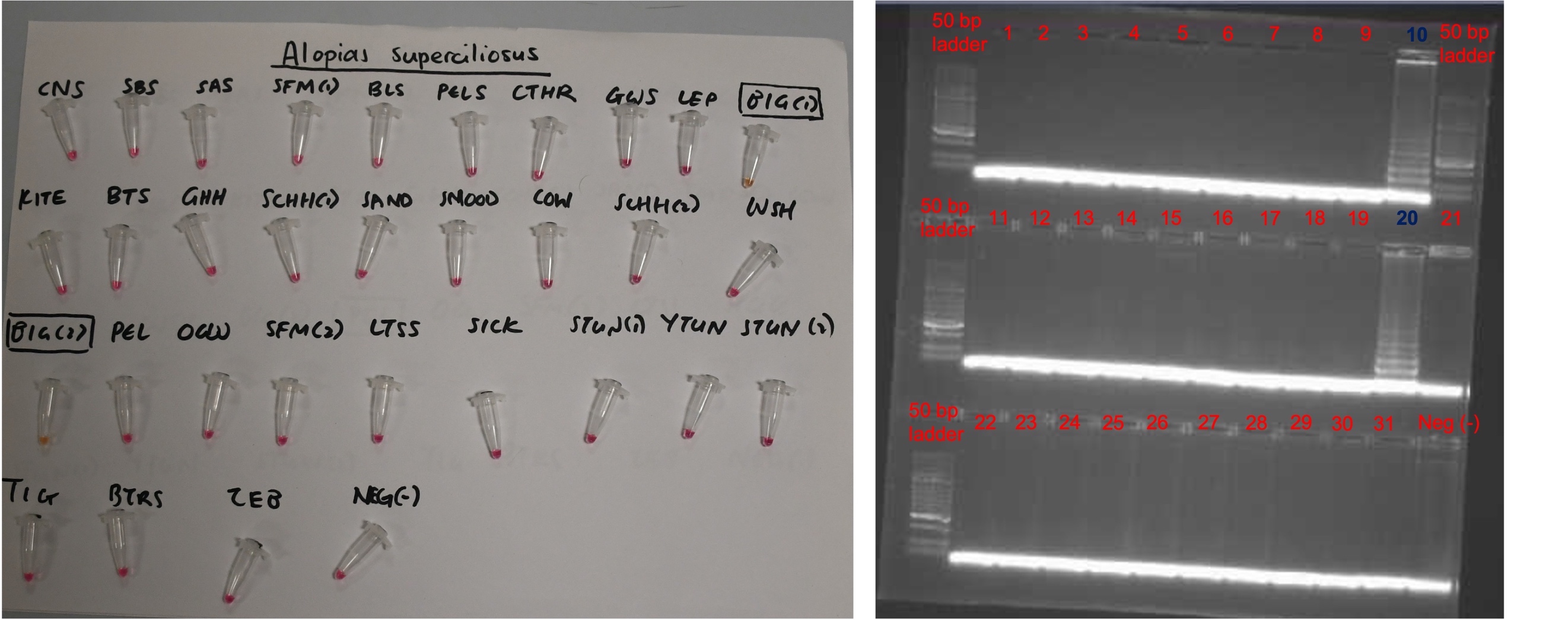


Figure S4 Primer specificity for target species bigeye thresher shark against 26 different species of elasmobranchs and fish (Table 1). (a) Showing LAMP colour change with target and non-target species in order from top right to bottom left and (b) gel image showing LAMP amplification of same species (numbered 1 – 31); red = non-target species, dark blue = target species: (1) CNS = clearnose skate (*Raja eglanteria*), (2) SBS = sandbar shark (*Carcharhinus plumbeus*), (3) SAS = salmon shark (*Lamna ditropis*), (4) SFM (1) = shortfin mako shark (*Isurus oxyrinchus*), (5) BLS = blue shark (*Prionace glauca*), (6) PELS = pelagic stingray (*Pteroplatyrygon violacea*), (7) CTHR = common thresher shark (*Alopias vulpinas*), (8) GWS = great white shark (*Carcharodon carcharias*), (9) LEP = leopard shark (*Triakis semifasciata*), (10) BIG (1) bigeye thresher shark (*A. superciliosus*), (11) KITE = kitefin shark (*Dalatis longa*), (12) BTS = black tip shark (*Carcharhinus limbatus*), (13) GHH = great hammerhead (*Sphyrna mokarran*), (14) SCHH (1) = scalloped hammerhead (*S. lewini*), (15) SAND = sand tiger shark (*Carcharias taurus*), (16) SMOOD = smooth dogfish (*Mustelus canis*), (17) COW = cownose ray (*Rhinoptera bonasus*), (18) SCHH (2), (19) WSH = whale shark (*Rhincodon typus*), (20) BIG (2), (21) PEL = pelagic thresher shark (*A. pelagicus*), (22) OCW = oceanic whitetip (*Carcharhinus longimanus*), (23) SFM (2), (24) LTSS = long tail stingray (*Dasyatis longa*), (25) SICK = sicklefin smoothhound (*M. lunulatus*), (26) STUN (1) skipjack tuna (*Katsuwonus pelamis*), (27) YTUN = yellowfin tuna (*Thunnus albacores*), (28) STUN (2), (29) TIG = tiger shark (*Galeocerdo cuvier*), (30) BTRS = black tip reef shark (*C. melanopterus*), (31) ZEB = zebra shark (*Stegastoma fasciatum*) and Neg (-) = negative control.

**
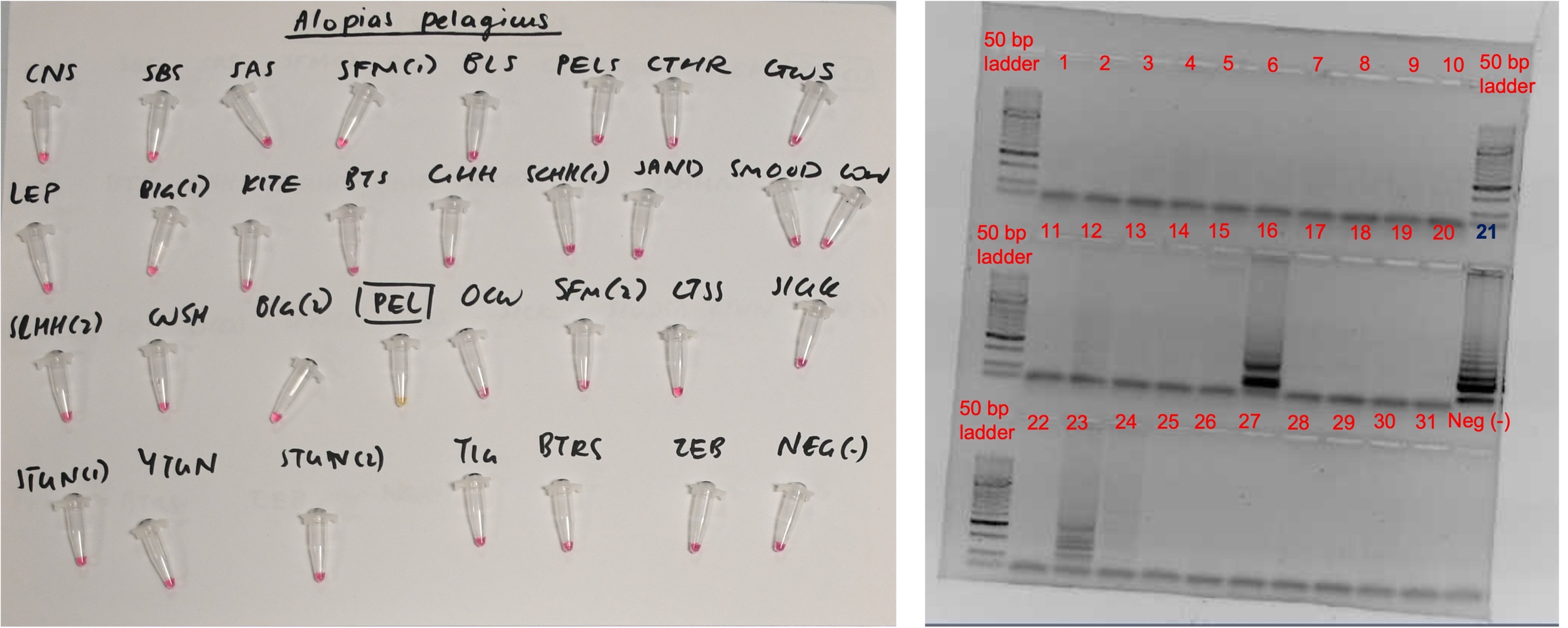
**

**a)**

**b)**

Figure S5 Primer specificity for target species pelagic thresher shark against 26 different species of elasmobranchs and fish (Table 1). (a) Showing LAMP colour change with target and non-target species in order from top right to bottom left and (b) gel image showing LAMP amplification of same species (numbered 1 – 31); red = non-target species, dark blue = target species: (1) CNS = clearnose skate (*Raja eglanteria*), (2) SBS = sandbar shark (*Carcharhinus plumbeus*), (3) SAS = salmon shark (*Lamna ditropis*), (4) SFM (1) = shortfin mako shark (*Isurus oxyrinchus*), (5) BLS = blue shark (*Prionace glauca*), (6) PELS = pelagic stingray (*Pteroplatyrygon violacea*), (7) CTHR = common thresher shark (*Alopias vulpinas*), (8) GWS = great white shark (*Carcharodon carcharias*), (9) LEP = leopard shark (*Triakis semifasciata*), (10) BIG (1) bigeye thresher shark (*A. superciliosus*), (11) KITE = kitefin shark (*Dalatis longa*), (12) BTS = black tip shark (*Carcharhinus limbatus*), (13) GHH = great hammerhead (*Sphyrna mokarran*), (14) SCHH (1) = scalloped hammerhead (*S. lewini*), (15) SAND = sand tiger shark (*Carcharias taurus*), (16) SMOOD = smooth dogfish (*Mustelus canis*), (17) COW = cownose ray (*Rhinoptera bonasus*), (18) SCHH (2), (19) WSH = whale shark (*Rhincodon typus*), (20) BIG (2), (21) PEL = pelagic thresher shark (*A. pelagicus*), (22) OCW = oceanic whitetip (*Carcharhinus longimanus*), (23) SFM (2), (24) LTSS = long tail stingray (*Dasyatis longa*), (25) SICK = sicklefin smoothhound (*M. lunulatus*), (26) STUN (1) skipjack tuna (*Katsuwonus pelamis*), (27) YTUN = yellowfin tuna (*Thunnus albacores*), (28) STUN (2), (29) TIG = tiger shark (*Galeocerdo cuvier*), (30) BTRS = black tip reef shark (*C. melanopterus*), (31) ZEB = zebra shark (*Stegastoma fasciatum*) and Neg (-) = negative control.


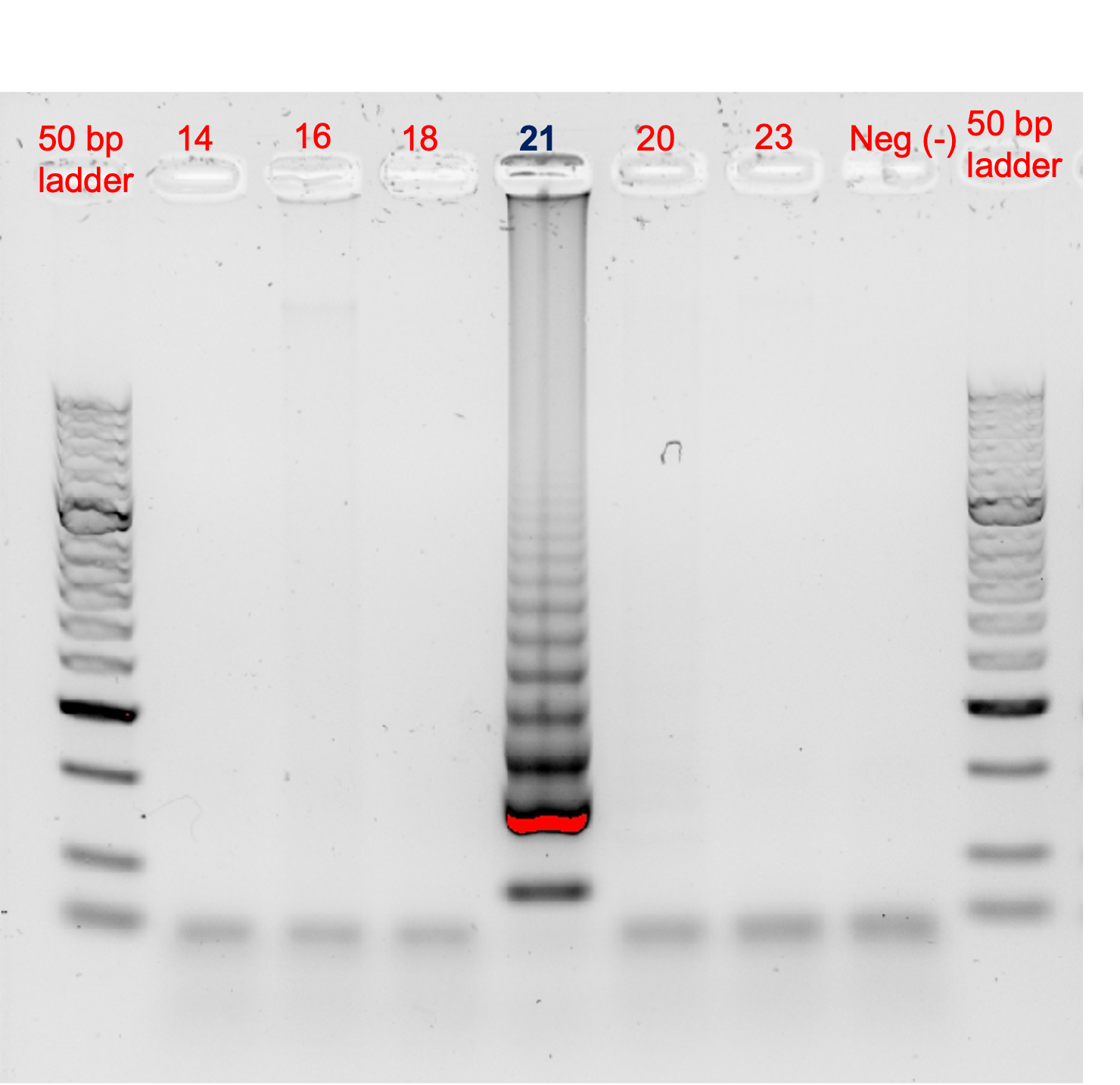


Figure S6 Repeat assay for pelagic thresher shark against five non-target species (as there was contamination in gels): 14 = scalloped hammerhead (*Sphyrna lewini*), 16 = smooth dogfish (*Mustelus canis*), 18 = scalloped hammerhead, 21 = pelagic thresher (*Alopias pelagicus*), 20 = bigeye thresher (*A. superciliosus*), 23 = shortfin mako shark (*Isurus oxyrinchus*) and Neg (-) = negative control, where red = non-target species and dark blue = target species.


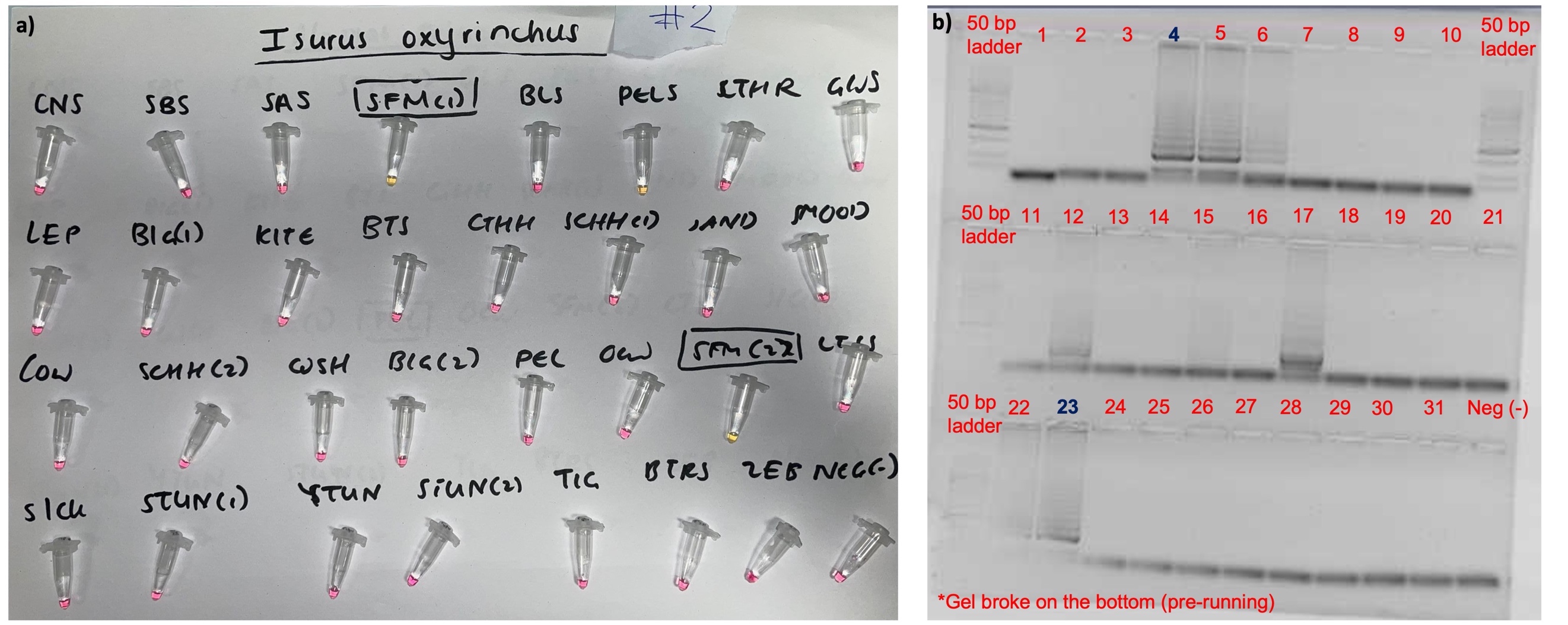


Figure S7 Primer specificity for target species shortfin mako shark against 26 different species of elasmobranchs and fish (Table 1). (a) Showing LAMP colour change with target and non-target species in order from top right to bottom left and (b) gel image showing LAMP amplification of same species (numbered 1 – 31); red = non-target species, dark blue = target species: (1) CNS = clearnose skate (*Raja eglanteria*), (2) SBS = sandbar shark (*Carcharhinus plumbeus*), (3) SAS = salmon shark (*Lamna ditropis*), (4) SFM (1) = shortfin mako shark (*Isurus oxyrinchus*), (5) BLS = blue shark (*Prionace glauca*), (6) PELS = pelagic stingray (*Pteroplatyrygon violacea*), (7) CTHR = common thresher shark (*Alopias vulpinas*), (8) GWS = great white shark (*Carcharodon carcharias*), (9) LEP = leopard shark (*Triakis semifasciata*), (10) BIG (1) bigeye thresher shark (*A. superciliosus*), (11) KITE = kitefin shark (*Dalatis longa*), (12) BTS = black tip shark (*Carcharhinus limbatus*), (13) GHH = great hammerhead (*Sphyrna mokarran*), (14) SCHH (1) = scalloped hammerhead (*S. lewini*), (15) SAND = sand tiger shark (*Carcharias taurus*), (16) SMOOD = smooth dogfish (*Mustelus canis*), (17) COW = cownose ray (*Rhinoptera bonasus*), (18) SCHH (2), (19) WSH = whale shark (*Rhincodon typus*), (20) BIG (2), (21) PEL = pelagic thresher shark (*A. pelagicus*), (22) OCW = oceanic whitetip (*Carcharhinus longimanus*), (23) SFM (2), (24) LTSS = long tail stingray (*Dasyatis longa*), (25) SICK = sicklefin smoothhound (*M. lunulatus*), (26) STUN (1) skipjack tuna (*Katsuwonus pelamis*), (27) YTUN = yellowfin tuna (*Thunnus albacores*), (28) STUN (2), (29) TIG = tiger shark (*Galeocerdo cuvier*), (30) BTRS = black tip reef shark (*C. melanopterus*), (31) ZEB = zebra shark (*Stegastoma fasciatum*) and Neg (-) = negative control.


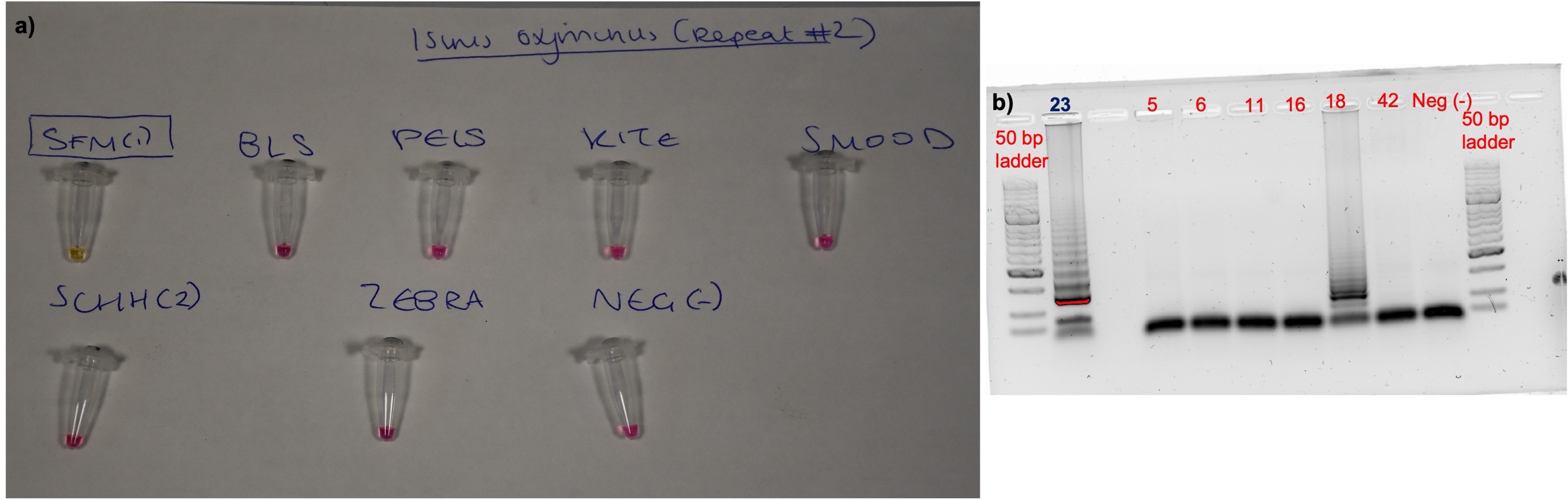


Figure S8 Repeat assay for shortfin mako shark against seven non-target species (as there was contamination in colour changes and gels): LAMP showing colour change (a) and (b) gel for shortfin mako shark against seven non-target species in order: (23) SFM = shortfin mako shark (*Isurus oxyrinchus*), (5) BLS = blue shark (Prionace glauca), (6) PELS = pelagic stingray (*Pteroplatyrygon violacea*), (11) KITE = kitefin shark (*Dalatis licha*), (16) SMOOD = smooth dogfish (*Mustelus canis*), (18) SCHH = scalloped hammerhead (*Sphyrna lewini*), (42) ZEBRA = zebra shark (*Stegastoma fasciatum*) and Neg (-) = negative control, where red = non-target species and dark blue = target species.

**SI 4: Integrated LOC**

LOCs were tested against the three target species of sharks (bigeye and pelagic thresher, and shortfin mako shark) and one non-target species of shark (blue shark (Prionace glauca)) in the lab (*n* ≥ 3) (Figure S9). Please note that although these pictures look grey, they were photographed on a white piece of A4 paper as background.


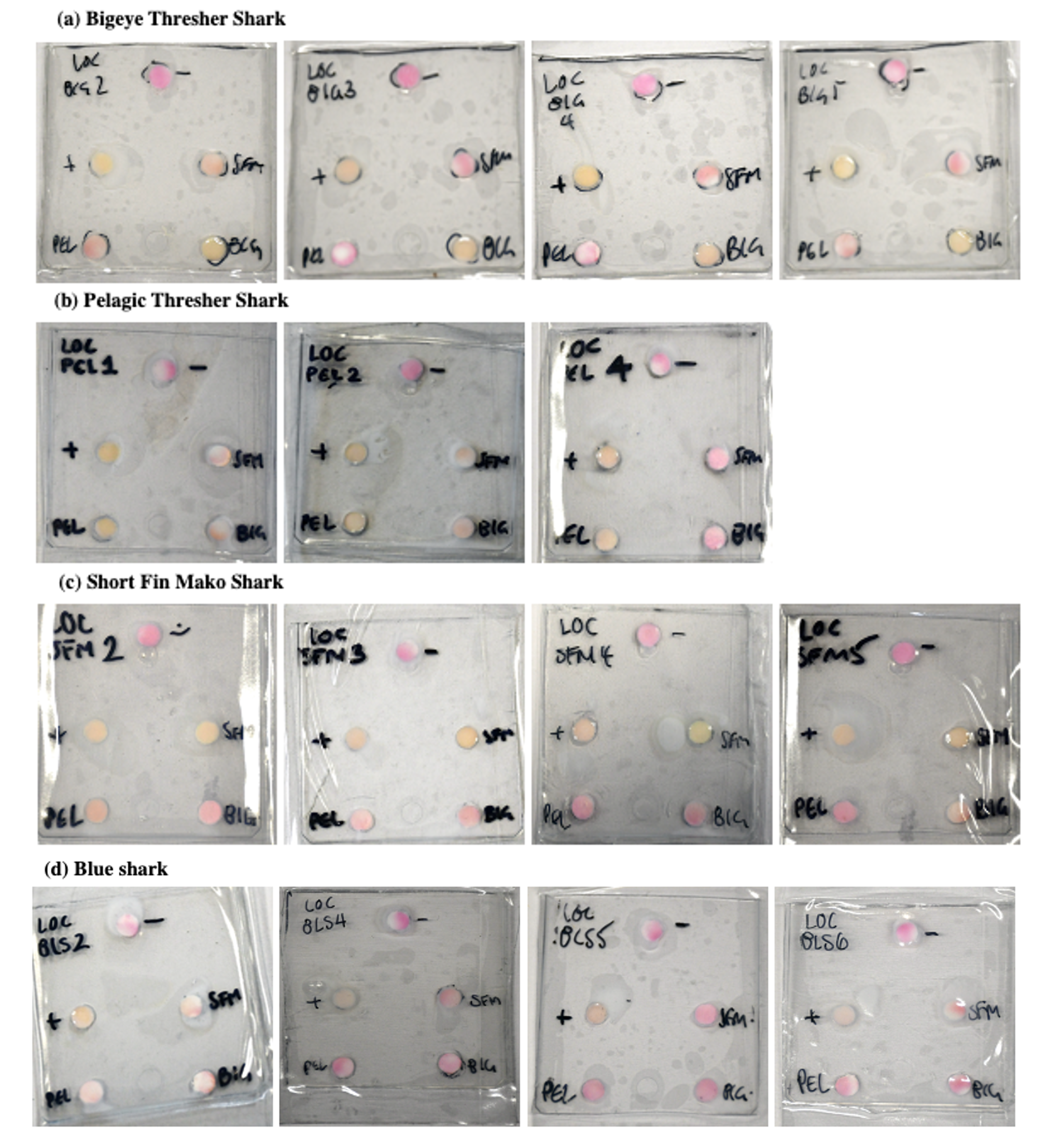
**Figure S9 Successful LOCs for target CITES-species where yellow indicates amplification and pink indicates no amplification.**


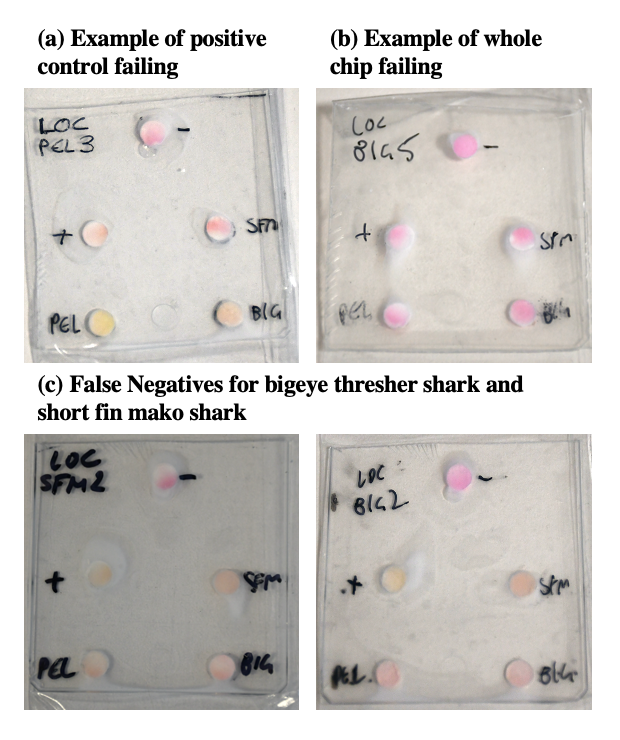


Figure S10 Examples of LOCs that did not work, (a) where either the positive control did not change, (b) where the whole chip failed or (c) the target species did not change colour, but the positive control did (False Negative) for bigeye thresher and shortfin mako shark (SFM 2, left panel) and bigeye thresher (BIG 2, right panel).

**Cost comparison with conventional methods**

Cost comparisons were based on prices in the UK in 2023 and therefore these are just an estimation for comparison as costs and availability of reagents in different places and countries with differ and be hard to quantify. Costs of the DNA Barcoding method were based on Field et al., (2015) [9]. The cost to carry out one LOC is $6.00 per sample (Table S3; LOC) and for DNA Barcoding is $13.3 (Table S3; DNA Barcoding). The cost breakdown for the DNA barcoding is prior to any upstream processing/analysis such as Sangar Sequencing which would increase the cost further.

Table S 3: Cost breakdown of one Lab-on-a-Chip (LOC) (this study) and one sample for DNA barcoding based on UK prices in 2023. *The exchange rate of 1 GBP = 1.27 USD was used to covert UK prices to USD.*

*NA = Six small filters were cut from one Whatman © GF/C Filter (one filter cut out = £0.039). Six are needed for one LOC.*

**References**

[1] Shaw KJ, Joyce DA, Docker PT, Dyer CE, Greenman J, Greenway GM, et al. Simple practical approach for sample loading prior to DNA extraction using a silica monolith in a microfluidic device. Lab Chip. 2009;9:3430–2.

[2] Shaw KJ, Thain L, Docker PT, Dyer CE, Greenman J, Greenway GM, et al. The use of carrier RNA to enhance DNA extraction from microfluidic-based silica monoliths. Anal Chim Acta. 2009;652:231–3.

[3] Montgomery GW, Sise JA. Extraction of dna from sheep white blood cells. New Zeal J Agric Res. 1990;33(3):437–41.

[4] Tian H, Hühmer AFR, Landers JP. Evaluation of silica resins for direct and efficient extraction of dna from complex biological matrices in a miniaturized format. Anal Biochem. 2000;283:175–91.

[5] Kashkary L, Kemp C, Shaw KJ, Greenway GM, Haswell SJ. Improved DNA extraction efficiency from low level cell numbers using a silica monolith based micro fluidic device. Anal Chim Acta [Internet]. 2012;750:127–31. Available from: http://dx.doi.org/10.1016/j.aca.2012.05.019

[6] Mosley O, Melling L, Tarn MD, Kemp C, Esfahani MMN, Pamme N, et al. Sample introduction interface for on-chip nucleic acid-based analysis of: Helicobacter pylori from stool samples. Lab Chip. 2016;16:2108–15.

[7] R Core Team. R: A language and environment for statistical computing. R Foundation for Statistical Computing. 2019.

[8] Giraudoux P. pgirmess: Spatial Analysis and Data Mining for Field Ecologists [Internet]. 2018. Available from: https://cran.r-project.org/package=pgirmess

[9] Fields AT, Abercrombie DL, Eng R, Feldheim K, Chapman DD. A novel mini-DNA barcoding assay to identify processed fins from internationally protected shark species. PloS one. 2015; 10(2): e0114844.
